# Determinants of FIV and HIV Vif sensitivity of feline APOBEC3 restriction factors

**DOI:** 10.1186/s12977-016-0274-9

**Published:** 2016-07-01

**Authors:** Zeli Zhang, Qinyong Gu, Ananda Ayyappan Jaguva Vasudevan, Anika Hain, Björn-Philipp Kloke, Sascha Hasheminasab, Daniel Mulnaes, Kei Sato, Klaus Cichutek, Dieter Häussinger, Ignacio G. Bravo, Sander H. J. Smits, Holger Gohlke, Carsten Münk

**Affiliations:** Clinic for Gastroenterology, Hepatology, and Infectiology, Medical Faculty, Heinrich-Heine-University Düsseldorf, Building 23.12.U1.82, Moorenstr. 5, 40225 Düsseldorf, Germany; Department of Medical Biotechnology, Paul-Ehrlich-Institute, Paul-Ehrlich-Str. 51-59, 63225 Langen, Germany; Institute of Biochemistry, Heinrich Heine University Düsseldorf, Universitätsstr. 1, 40225 Düsseldorf, Germany; Institute of Pharmaceutical and Medicinal Chemistry, Heinrich Heine University Düsseldorf, Universitätsstr. 1, 40225 Düsseldorf, Germany; Laboratory of Viral Pathogenesis, Institute for Virus Research, Kyoto University, Kyoto, 6068507 Japan; CREST, Japan Science and Technology Agency, Saitama, 3220012 Japan; MIVEGEC (UMR CNRS 5290, IRD 224, UM), National Center of Scientific Research (CNRS), 34394 Montpellier, France; BioNTech RNA Pharmaceuticals GmbH, An der Goldgrube 12, 55131 Mainz, Germany

**Keywords:** APOBEC3, FIV, Gene evolution, HIV, Homeobox, Homology modelling, Restriction factor, SIV, Vif

## Abstract

**Background:**

Feline immunodeficiency virus (FIV) is a global pathogen of Felidae species and a model system for Human immunodeficiency virus (HIV)-induced AIDS. In felids such as the domestic cat (*Felis catus*), APOBEC3 (A3) genes encode for single-domain A3Z2s, A3Z3 and double-domain A3Z2Z3 anti-viral cytidine deaminases. The feline A3Z2Z3 is expressed following read-through transcription and alternative splicing, introducing a previously untranslated exon in frame, encoding a domain insertion called linker. Only A3Z3 and A3Z2Z3 inhibit Vif-deficient FIV. Feline A3s also are restriction factors for HIV and Simian immunodeficiency viruses (SIV). Surprisingly, HIV-2/SIV Vifs can counteract feline A3Z2Z3.

**Results:**

To identify residues in feline A3s that Vifs need for interaction and degradation, chimeric human–feline A3s were tested. Here we describe the molecular direct interaction of feline A3s with Vif proteins from cat FIV and present the first structural A3 model locating these interaction regions. In the Z3 domain we have identified residues involved in binding of FIV Vif, and their mutation blocked Vif-induced A3Z3 degradation. We further identified additional essential residues for FIV Vif interaction in the A3Z2 domain, allowing the generation of FIV Vif resistant A3Z2Z3. Mutated feline A3s also showed resistance to the Vif of a lion-specific FIV, indicating an evolutionary conserved Vif–A3 binding. Comparative modelling of feline A3Z2Z3 suggests that the residues interacting with FIV Vif have, unlike Vif-interacting residues in human A3s, a unique location at the domain interface of Z2 and Z3 and that the linker forms a homeobox-like domain protruding of the Z2Z3 core. HIV-2/SIV Vifs efficiently degrade feline A3Z2Z3 by possible targeting the linker stretch connecting both Z-domains.

**Conclusions:**

Here we identified in feline A3s residues important for binding of FIV Vif and a unique protein domain insertion (linker). To understand Vif evolution, a structural model of the feline A3 was developed. Our results show that HIV Vif binds human A3s differently than FIV Vif feline A3s. The linker insertion is suggested to form a homeo-box domain, which is unique to A3s of cats and related species, and not found in human and mouse A3s. Together, these findings indicate a specific and different A3 evolution in cats and human.

**Electronic supplementary material:**

The online version of this article (doi:10.1186/s12977-016-0274-9) contains supplementary material, which is available to authorized users.

## Background

APOBEC3 (A3) cytidine deaminases are anti-viral restriction factors containing either one or two zinc (Z)-binding domains found in different clade-specific gene numbers and gene arrangements in placental mammals [1–4]. For example, primates have seven genes (A3A–A3D, A3F–A3H), while cats encode four genes (A3Z2a–A3Z2c, A3Z3) [3, 5]. These A3 proteins target broadly viruses and mobile genetic elements that depend on reverse transcription, but also show antiviral activity against unrelated viruses (for recent reviews see [6, 7]). Some retroviruses express viral A3-counteracting proteins, such as Vif of lentiviruses, Bet of foamy viruses, the nucleocapsid of *Human T cell leukemia virus type 1* (HTLV-1), and the glycosylated (glyco)-Gag of *Murine leukemia virus* (MLV) [8–13]. The Vif protein prevents encapsidation of host-cell derived A3 proteins into nascent viral particles. In the absence of Vif, encapsidated A3s inhibit lentiviruses during infection by deamination of cytidines in the single-stranded DNA formed during reverse transcription, by introducing G-to-A mutations in the coding strand. Additionally, some A3s inhibit virus replication by reducing reverse transcription and integration via non-editing mechanisms [14–19].

The domestic cat *Felis catus* (Fca) is the host to many diverse retroviruses, such as the lentivirus *Feline immunodeficiency virus* (FIV), gammaretroviruses of the *Feline leukemia virus* (FeLV) group, and the spumaretrovirus *Feline foamy virus* (FFV) (for reviews see [20–23]). In a small proportion of naturally infected domestic cats, FIV causes an immunodeficiency disease similar to *Human immunodeficiency virus type 1* (HIV-1)-induced AIDS [24]. However, highly pathogenic FIV isolates can cause mortality up to 60 % under experimental conditions [25–27]. Thus, FIV infection of cats is a valuable animal model to study HIV-1 and AIDS [28–30]. In addition to the domestic cat, species-specific FIVs that might cause disease in some natural hosts have been isolated in many *Felidae* [31]. FFVs replicate in domestic cats and in other *Felidae* and are not causing disease [32–34]. In contrast, FeLVs are pathogenic and induce in domestic cats serious diseases such as lymphomas and anemia [24], but are rarely found in other *Felidae* [31].

**Fig. 1:**
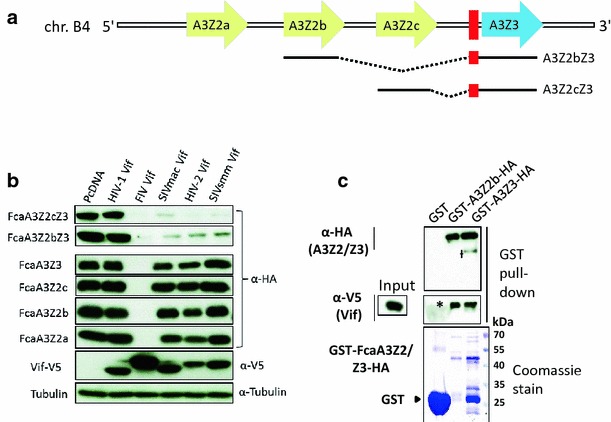
The interaction of feline APOBEC3s with FIV Vif. **a** Representation of APOBEC3 (A3) genes in the genome of *Felis catus*, chromosome (chr.) B4. Coding regions of the A3 genes (A3Z2a, A3Z2b, A3Z2c, A3Z3) shown as *arrows*. *Red rectangle* exon 2 of A3Z3 that is untranslated in the A3Z3 mRNA, however translated (“linker domain”) in readthrough transcripts A3Z2bZ3 and A3Z2cZ3. Spliced-out introns (*dashed lines*) are indicated, mRNAs for A3Z2s and A3Z3 not shown. For details, see references [4, 36]. **b** 293T cells were transfected with expression plasmids for FcaA3Z2a, FcaA3Z2b, FcaA3Z2c, FcaA3Z3, FcaA3Z2bZ3 and FcaA3Z2cZ3 together with HIV-1, SIVmac, HIV-2 and SIVsmm or FIV Vif or no Vif (replaced by pcDNA3.1). FcaA3s, Vifs and tubulin were visualized by immunoblot using anti-HA, anti-V5 and anti-tubulin antibodies. **c** Immunoprecipitation of *E. coli* expressed FcaA3Z2 and -Z3 (GST fusion proteins) after mixing with 293T-derived FIV Vif (Vif-V5). *Asterisk* indicates the impression of GST blob on the blot and *Dagger* denotes for a possible degradation product of FcaA3Z3

The domestic cat, and likely all other *Felidae*, encode four A3 genes, three closely related A3Z2 genes (A3Z2a, A3Z2b, A3Z2c) and one A3Z3 gene [4, 35]. Besides the four canonical A3 proteins, the cat genome can express, by read-through transcription and alternative splicing, a fifth A3 protein, namely the double-domain A3Z2Z3, with two detected variants A3Z2bZ3 and A3Z2cZ3 (Fig. 1a). A3Z2Z3s are also found in big cats (Pantherinae), indicating evolutionary conserved gene regulation [4, 36]. FIV Vif induces proteasome-dependent degradation of feline A3Z2s, A3Z3, and A3Z2Z3 [4, 37]. The double-domain feline A3Z2Z3 contains two FIV Vif interaction regions, one in each Z-domain [36]. Interestingly, and currently unexplained, FIVΔ*vif* can be inhibited by feline AZ3 and A3Z2Z3, but not by A3Z2s [4, 36]. A reverse observation was made with FFVΔ*bet*, where feline A3Z2s act as major inhibitors while A3Z3 and A3Z2Z3 only moderately reduce the infectivity of FFVΔ*bet* [4, 10, 38, 39]. Recent data indicate that certain polymorphisms in feline A3Z3 genes correlate with the susceptibility to FIV and/or FeLV infections [40].

FIV Vif induces the poly-ubiquitination of feline A3s and bridges A3s to an E3 ubiquitin ligase complex containing Cullin5 (Cul5), Elongin B/C (EloB/C), and RING-box protein RBX2 [37]; HIV-1 Vif forms a similar E3-ligase complex [41–43]. However, while HIV-1 Vif needs to additionally interact with the CBF-β protein to be stabilized and form this multiprotein complex [44, 45], FIV Vif does not bind CBF-β, and the FIV Vif-induced degradation of feline A3s does not require CBF-β to be expressed [46–49]. HIV-1 Vif cannot counteract feline A3s, and HIV-1 is therefore inhibited to various degrees by all feline A3s, with A3Z2Z3 displaying the strongest anti-HIV activity [36, 50–52]. The mechanistic reason preventing HIV-1 Vif from degrading feline A3s is unclear, especially because HIV-1 Vif and feline A3Z2Z3 are recovered together using co-immunoprecipitation assays [51]. In contrast to the Vif protein of HIV-1, Vif of Simian immunodeficiency virus from macaques (SIVmac) induces degradation of feline A3s [46, 51]. To assess the feasibility of generating an animal model for the human system based on FIV, we and others cloned FIV *vif* into HIV-1 and proved that in feline cell lines the A3 proteins are the dominant restriction factors against HIV-1 [36, 51].

In order to understand the FIV Vif interaction with feline A3 proteins, we identified in this study important A3 residues and used a homology model of feline A3Z2Z3 to describe the structure–function relationship of these potential FIV Vif binding amino acids.

## Results

### FIV and HIV-2/SIVmac/smm Vif induced degradation of felines A3s

In order to identify the molecular interaction of the FIV Vif protein and feline A3 proteins, we used FIV of domestic cats (*Felis catus*, Fca), hereafter referred as FIV. Co-transfection experiments of cat-derived A3s and FIV Vif expression plasmids were performed in 293T cells. All A3 constructs expressed the corresponding A3 protein as a C-terminal HA-tag, whereas Vif was expressed as a C-terminal V5-tag fusion protein. In addition, we also studied Vifs derived from HIV-1, HIV-2, SIVmac, and SIVsmm. Immunoblots of protein extracts from cells co-expressing both A3 and Vif were used as a read-out for degradation of the respective A3 protein. Results in Fig. 1b show that FIV Vif induces degradation of single-domain feline A3Z2a, A3Z2b, A3Z2c, A3Z3, and double-domain A3Z2bZ3 and A3Z2cZ3 in agreement with previous reports [4, 36, 37, 51]. The double-domain feline A3Z2bZ3 and A3Z2cZ3 were degraded by SIVmac Vif as seen before [46, 51], as well by the Vifs of SIVsmm and HIV-2. For subsequent experiments we used the expression plasmid FcaA3Z2bZ3, hereafter referred to as feline A3Z2Z3 for simplicity.

To understand, whether FIV Vif binds directly to feline A3s, we expressed A3Z2 and A3Z3 as GST fusion proteins in *E. coli*. Recombinant A3s were purified by affinity chromatography and mixed with lysates of 293T cells expressing FIV Vif. Following GST pulldown, immunoblots showed Vif binding to GST-A3Z2 and to GST-A3Z3 but not to GST (Fig. 1c). We further explored the interaction of FIV Vif with feline A3s by analyzing the cellular distribution in co-expressing cells. HOS cells were transfected either with plasmids encoding for feline A3Z2, A3Z3, or A3Z2Z3 alone or together with a plasmid encoding for FIV Vif-TLQAAA. The TLQ to AAA mutation in the Vif putative BC-box prevents its interaction with the E3 complex [37]. Feline A3 proteins showed a mostly cytoplasmic localization with no or very little nuclear A3, and feline A3Z3 localized in addition to the nucleoli (Additional file 1: Fig. S1, compare to Fig. S4). Nucleolar localization of A3Z3 proteins derived from humans and horses had been described before [53]. Very similar to the A3s, FIV Vif-TLQAAA showed a cytoplasmic distribution with little presence in the nucleus. Under these experimental conditions, strong co-localization of Vif and A3s was detected in cytoplasmic areas near the nucleus (Additional file 1: Fig. S1).

### Identification of feline A3Z3 residues important for FIV Vif induced degradation

**Fig. 2:**
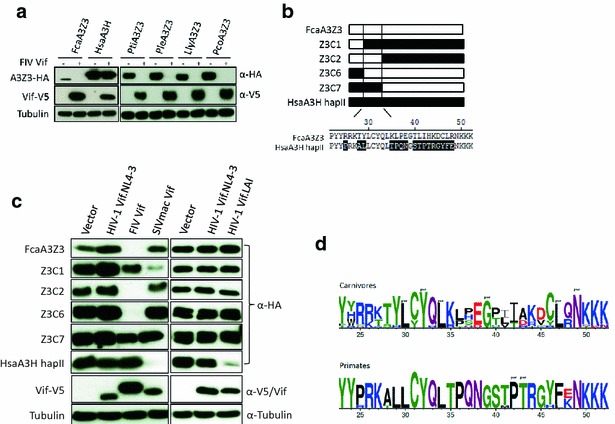
Identification of residues in FcaA3Z3 for FIV Vif induced degradation. **a** Expression plasmids for FcaA3Z3, HsaA3H and big cat A3Z3s were co-transfected with FIV Vif into 293T cells. Cell lysates were analyzed by immunoblot. A3s contain a C-terminal HA-tag, Vifs contain a C-terminal V5-tag. FcaA3s represent domestic cat (*Felis catus*) APOBEC3s. Pti, Ple, Lly and Pco represent *Panthera tigris corbetti*; *Panthera leo bleyenberghi*; *Lynx lynx*; *Puma concolor*. **b** Scheme of FcaA3Z3/HsaA3H.hapII chimeras (Z3C1, -C2, –C6, -C7). *Highlighted* sequence diversity between HsaA3H and FcaA3Z3 in an N-terminal region. **c** 293T cells were co-transfected with expression plasmids for FcaA3Z3, Z3C1, Z3C2, Z3C6, Z3C7 or HsaA3H hapII and FIV Vif, HIV-1 (NL4-3 or LAI) or SIVmac Vif. The expression of chimeras and Vif proteins were detected by using anti-HA and anti-V5 antibodies, respectively. Tubulin served as loading control. **d** Amino acid logo for the N-terminus in 15 A3Z3 sequences from ten Carnivores species (*upper panel*) and for eight A3Z3 sequences from eight Primates species (*lower panel*). Residues identified to evolve under purifying selection are labelled with “pur”. No residue was identified to evolve under diversifying selection in this A3Z3 stretch

Feline A3Z3 and A3Z2Z3 are the restriction factors for FIVΔ*vif*, whereas A3Z2s are not active against FIVΔ*vif* [4, 36, 37, 51]. To characterize the Vif interaction with residues in feline A3Z3, A3Z3s derived from humans (A3H haplotype II, HsaA3H) and big cats (tiger, *Panthera tigris*, Pti; lion, *Panthera leo*, Ple; lynx, *Lynx lynx*, Lly; puma, *Puma concolor*, Pco) (protein alignments are highlighted in Additional file 1: Fig. S2) were used in co-transfection experiments with FIV Vif. A3s derived from tiger, lion, lynx, and puma were efficiently degraded by FIV Vif (Fig. 2a). Because A3H was resistant to FIV Vif-induced degradation, the construction of Hsa–Fca chimeric A3Z3s promised a rational approach to identify the A3Z3/FIV-Vif binding region. The chimeras Z3C1 and Z3C2 spanned respectively amino acids 1–22 and 1–50 of feline A3Z3, with the remaining part being derived from A3H, whereas Z3C6 and Z3C7 were mostly feline A3Z3 with residues 1–22 or 1–50 derived from A3H (Fig. 2b). Among the four A3Z3 chimeras, Z3C2 and Z3C6 were efficiently degraded by FIV Vif, while Z3C1 and Z3C7 showed resistance to degradation (Fig. 2c). HIV-1 Vif (derived from clones NL4-3 or LAI) could not degrade any of the A3Z3 chimeras, but LAI Vif degraded A3H as reported before [54], and SIVmac Vif degraded Z3C1 and A3H but not Z3C2, Z3C6 and Z3C7 (Fig. 2c).

Our findings indicate that feline-derived residues shared by Z3C2 and Z3C6 (positions 23–50) are essential for FIV Vif interaction. This A3 stretch contained a number of positions evolving exclusively under purifying selection, for both carnivores and for primates (Fig. 2d). Globally, diversity among A3Z3 from carnivores was higher than among the primates’ orthologs (respectively 0.24 ± 0.02 vs 0.054 ± 0.007, overall average pairwise nucleotide distance ± bootstrap standard error estimate) (Additional file 1: Fig. S3A). During this analysis, we identified for the first time duplicated A3Z3s in the same genome (i.e. in-paralogs [55]) retrieved from different lineages within Caniformia (Ursidae, the giant panda and the polar bear; Phocidae, the Weddell seal; and Odobenidae, the walrus) but we could neither identify the two A3Z3 in-paralogs in Canidae (dog) nor in Mustelidae (ferret) genomes. By contrast, in all Felidae genomes that we have screened we could only identify one of these in-paralogs (Additional file 1: Fig. S3A).

**Fig. 3:**
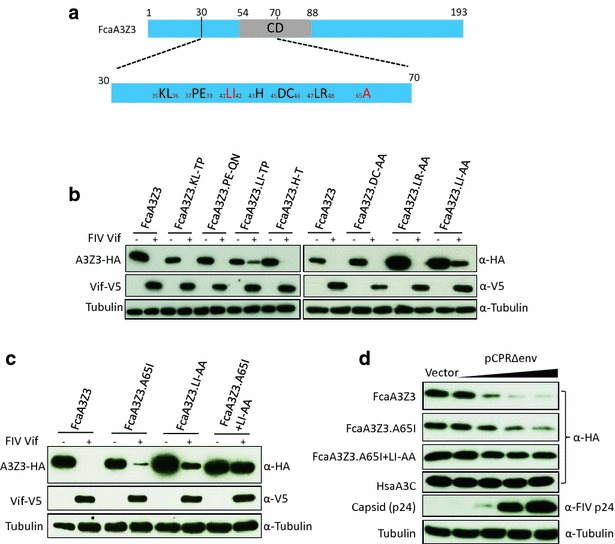
Generation of FIV Vif resistant FcaA3Z3. **a** Representation of FcaA3Z3 protein. Residues investigated are shown. *CD* cytidine deaminase domain. **b**, **c** Several mutants at N-terminal region of FcaA3Z3 were generated. To analyze the sensitivity of FcaA3Z3 mutants to FIV Vif, 293T cells were co-transfected with expression plasmids for FcaA3Z3 wild-type (FcaA3Z3) or indicated mutants and FIV Vif. 48 h later, FcaA3Z3, Vif and tubulin proteins were detected by immunoblot. **d** Increasing amounts of FIV *gag*-*pol*-*vif* helper plasmid pCPRΔ*env* that expresses virus-typical levels of FIV Vif without a tag were analyzed for degradation of HsaA3C, FcaA3Z3, FcaA3Z3.A65I and FcaA3Z3.A65I + LI-AA. 293T cells were co-transfected with 150 ng HsaA3C, FcaA3Z3, or A3Z3 mutants and pCPRΔ*env* (0, 10, 50, 150 or 250 ng). 48 h later, A3 expression was analyzed by immunoblots by using anti-HA antibody. Anti-FIV p24 detected expression of FIV helper plasmid pCPRΔ*env*, tubulin detection confirmed equal protein loading

The A3Z3 region position 23–50 differs in 16 amino acids between human and feline A3Z3s, and contains certain highly conserved amino acid positions (Fig. 2b, d). We mutated thus most feline-specific residues in feline A3Z3, in positions 35–38 and 40–48. Residues in position 35 + 36 (KL), 37 + 38 (PE), 41 + 42 (LI) and 43 (H) in A3Z3 were substituted by the corresponding ones found in A3H. Additionally, we exchanged the A3Z3 residues at position 45 + 46 (DC), 47 + 48 (LR) and 41 + 42 (LI) against AA (Fig. 3a). These mutated A3s were characterized for resistance to degradation by co-expression with FIV Vif. We found that only A3Z3s mutated at position 41 + 42 (LI ≫ TP and LI ≫ AA) showed partial resistance to degradation by Vif (Fig. 3b). A65I in feline A3Z3 has been described in Brazilian cats and discussed to be a relevant resistance mutation against FIV [40, 56]. Under our experimental conditions, A3Z3 mutated in position 65 (A65I) displayed only little resistance to Vif-mediated degradation (Fig. 3c). However, very important, the combination of mutations, A65I and L41A-I42A, resulted in an A3Z3 variant that showed complete resistance to FIV Vif degradation (Fig. 3c). We wondered whether experimental overexpression of the V5-tagged FIV Vif could mask the potency of the natural A65I variant to resist degradation. To address this question, we used as a source for Vif expression the replication-deficient FIV packaging construct pCPRΔ*env* [57]. Expression of increasing levels of pCPRΔ*env* in the presence of constant amounts of A3 revealed that the A65I mutation was degraded less efficiently than the wild-type A3Z3 (Fig. 3d). As a control we used A3C and A3Z3.A65I + LI-AA, which both showed no degradation by Vif derived by pCPRΔ*env*. Together, these findings indicate that the A65I mutation in feline A3Z3 mediates a partial protection, and that a combination with L41A-I42A resulted in enhanced resistance to Vif.

The stretch involved in the interaction with Vif encompassed a number of highly conserved residues between A3Z3s from carnivores and primates, as well as residues under purifying selection (Fig. 2d). The L41–I42 residues in cat A3Z3 identified to interact with Vif are strictly conserved (L|I) in A3Z3 from felids, to the extent that even the codons used are also strictly conserved (CTT|ATT) for the five Felidae species analyzed. Interestingly, the two A3Z3 paralogs in Caniformia display different amino acid profiles in this Vif-binding region (Additional file 1: Fig. S3A), and albeit chemically related, amino acid residues in these positions are variable (I/L/V|I/T). Finally, this A3Z3 stretch is very different in the corresponding positions in A3Z3 from primates (T/M|P). Altogether, evolutionary relationships for these two residues could thus at least partly explain species-specificity of the interaction between felidae A3Z3 and FIV Vif, reflecting adaptation and specific targeting.

### Generation of a FIV Vif resistant feline A3Z2Z3

**Fig. 4:**
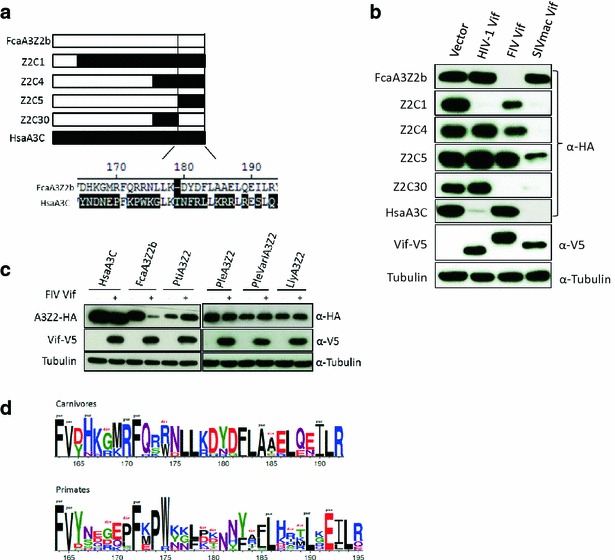
FIV Vif requires C-terminal FcaA3Z2 (A3Z2b) residues for A3 degradation. **a** Scheme of A3Z2/A3C chimeras (Z2C1, -C4, -C5, -C30). *Highlighted* are C-terminal sequence differences between FcaA3Z2 and HsaA3C. **b** 293T cells were co-transfected with expression plasmids for Z2C1, Z2C4, Z2C5 or Z2C30 and FIV Vif, HIV-1 or SIVmac Vif. A3s contain a C-terminal HA-tag; Vifs contain a C-terminal V5-tag. The expression of chimeras and Vif proteins were detected by using anti-HA and anti-V5 antibodies, respectively. Cell lysates were also analyzed for equal amounts of total proteins using the anti-tubulin antibody. **c** Expression plasmids for A3C, FcaA3Z2, and big cat A3Z2s were co-transfected with FIV Vif into 293T cells. 48 h later, cells were analyzed by immunoblot. The expression of A3 and Vif proteins were detected by using anti-HA and anti-V5 antibodies, respectively. FcaA3s represent domestic cat (*Felis catus*); Pti, Ple, and Lly represent *Panthera tigris corbetti*; *Panthera leo bleyenberghi*; *Lynx lynx, varI, variant I*. **d** Amino acid logo for the C-terminus in 17 A3Z2 sequences from 12 Carnivores species (*upper panel*) and for 51 A3Z2 sequences from 17 Primates species (*lower panel*). Residues identified to evolve under purifying or diversifying selection are respectively labelled with “pur” (*black*) or with “div” (*red*)

**Fig. 5:**
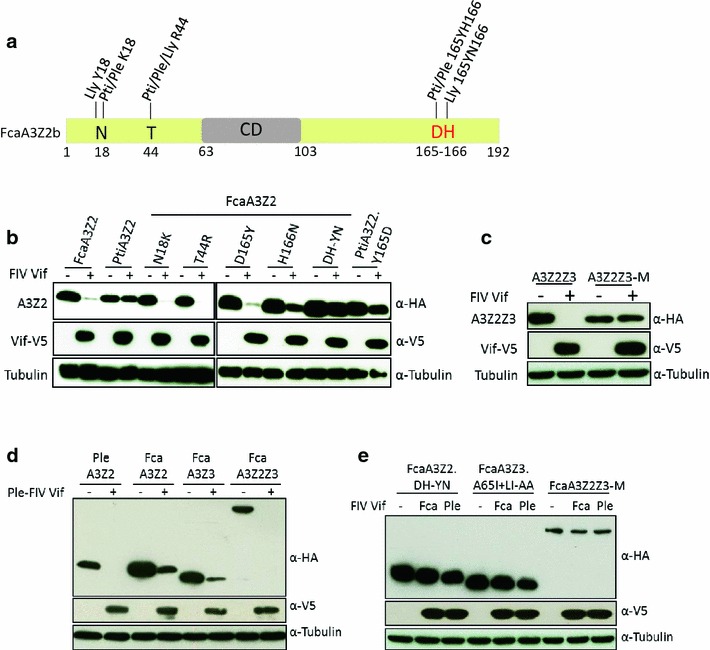
FcaA3Z2 and FcaA3Z2Z3 mutations block degradation by feline Vifs. **a** Representation of FcaA3Z2b protein. Residues investigated are shown. *CD* cytidine deaminase domain. Residues different found in A3Z2 of the domestic cat and big cats indicated. Pti, Ple, and Lly represent *Panthera tigris corbetti*; *Panthera leo bleyenberghi*; *Lynx lynx.* **b** Expression plasmids for FIV Vif and FcaA3Z2, PtiA3Z2 or several mutants of feline A3Z2 were co-transfected into 293T cells. The expression of A3 and Vif proteins were detected by using anti-HA and anti-V5 antibodies, respectively. **c** FcaA3Z2Z3-M that contains DH-YN and A65I + LI-AA mutations in Z2- and Z3-domains was analyzed. Expression plasmids for FcaA3Z2Z3 wild-type or FcaA3Z2Z3-M were transfected together with FIV Vif plasmid into 293T cells. 48 h later, immunoblots were used to detect the expression of A3 and Vif by anti-HA and anti-V5 antibodies, respectively. **d** Vif from lion-derived FIV (FIVple) degrades wild-type FcaA3s (from domestic cat) and PleA3Z2 (from lion). 293T cells were co-transfected with expression plasmids for wild-type FcaA3Z2, PleA3Z2, FcaA3Z3 and FcaA3Z2Z3 and FIVple Vif. 48 h later, cells were harvested and lysates were used for detecting the expression of Vif and A3s by anti-HA and anti-V5, respectively. Cell lysates were also analyzed for equal amounts of total proteins using the anti-tubulin antibody. **e** Expression plasmids for FcaA3s with mutations (FcaA3Z2.DH-YN, FcaA3Z3.A65I + LI-AA or FcaA3Z2Z3-M) were co-transfected with lion FIV Vif (FIVple Vif) or domestic cat FIV Vif (FIVfca Vif) and 48 h later, cell lysates were used for detecting FcaA3s, Vif, and tubulin by anti-HA, anti-V5 and anti-tubulin, respectively. Fca, *Felis catus*; Ple, *Panthera leo bleyenberghi*

Our results demonstrate that feline A3Z2 can also be efficiently degraded by FIV Vif, thus implying a specific interaction between both proteins (Fig. 1b). In order to generate an A3Z2Z3 protein resistant to FIV Vif, we decided to mutate as well the A3Z2 moiety. To identify residues important for FIV Vif interaction with feline A3Z2, chimeric A3s of A3Z2 and human A3C, called Z2C1, -C4, -C5 and -C30 (Fig. 4a), were co-expressed with FIV Vif. The chimeras Z2C1, Z2C4 and Z2C5 spanned the 1–22, 1–131 and 1–154 amino acids of feline A3Z2, respectively, the remaining parts being derived from A3C. Chimera Z2C30 was feline A3Z2, with amino acids 132–154 derived from A3C. Chimeras Z2C1 and Z2C4 showed moderately reduced protein levels when FIV Vif was co-expressed, chimera Z2C5 resistance to degradation, and chimera Z2C30 was efficiently degraded by FIV Vif (Fig. 4b). As controls, we investigated all chimeras for degradation by HIV-1 and SIVmac Vifs. HIV-1 Vif induced degradation of Z2C1 only, and SIVmac Vif completely degraded Z2C1, Z2C4 and Z2C30, and mostly Z2C5 (Fig. 4b). Because the Z2C5 chimera, in which the C-terminal 37 residues were of A3C origin, was resistant to FIV Vif, we speculated that the C-terminal region of cat A3Z2 could be important for FIV Vif-induced degradation. SIVmac Vif, which cannot degrade feline A3Z2 (Fig. 4b), interacts presumably with C-terminal human-derived sequences spanning A3C sequences present in Z2C5 and Z2C30 (Fig. 4b). In addition we analyzed the degradation sensitivity of A3Z2 proteins from big cats and found that FIV Vif did not induce degradation of A3Z2 from tiger, lion or lynx (Fig. 4c). These felid A3Z2s are very similar to FcaA3Z2 as they share 89–93 % identically conserved residues (Additional file 1: Figs. S2, S3B, Fig. 4d), whereas cat A3Z2 and human A3C are much more diverse and share only 47 % identical amino acids. Thus, we identified four positions in which all big cat A3Z2s differed from FcaA3Z2, in positions N18, T44, D165 and H166 (Additional file 1: Fig. S2, Fig. 5a). We mutated accordingly position 18 (N18K) and 44 (T44R) in FcaA3Z2, but found both mutants to be efficiently degraded by FIV Vif (Fig. 5b). Very similar, A3Z2.D165Y was depleted when co-expressed with FIV Vif. Interestingly, mutation of residue 166 (H166N) generated a partially Vif-resistant A3Z2 protein. We speculated that the adjacent D165 might enhance the Vif-resistance seen in the H166N variant. Indeed, the A3Z2.DH-YN mutant showed complete resistance to FIV Vif (Fig. 5b). We also analyzed tiger A3Z2.Y165D but could not reverse the resistance to degradation by FIV Vif (Fig. 5b). We conclude that D165-H166 in the C-terminal region of cat A3Z2 are important for Vif-mediated degradation together with other residues that remain to be characterized.

Finally, we constructed A3Z2Z3-M containing D165Y, H166N in Z2 and A65I + L41A, I42A in Z3. Co-expression experiments of A3Z2Z3-M with FIV Vif showed that this A3 variant was Vif-resistant (Fig. 5c). Importantly, the mutations that generated Vif-resistance did not impact the subcellular localization of the feline A3, as demonstrated by confocal microscopy of transiently transfected HOS cells (Additional file 1: Fig. S4). We also studied lion specific FIV (FIVple) Vif, which shares only 52 % identical residues with domestic cat FIV Vif (Additional file 1: Fig. S2C). FIVple Vif was able to induce degradation of PleA3Z2 and of FcaA3Z2, A3Z3 and A3Z2Z3 (Fig. 5d). Interestingly, FIVple Vif could not induce degradation of the mutated cat A3s A3Z2.DH-YN, A3Z3.A65I + LIAA and A3Z2Z3-M (Fig. 5e). These findings suggest that Vifs from lion and from domestic cat FIVs interact with identical residues in the domestic cat A3s.

**Fig. 6:**
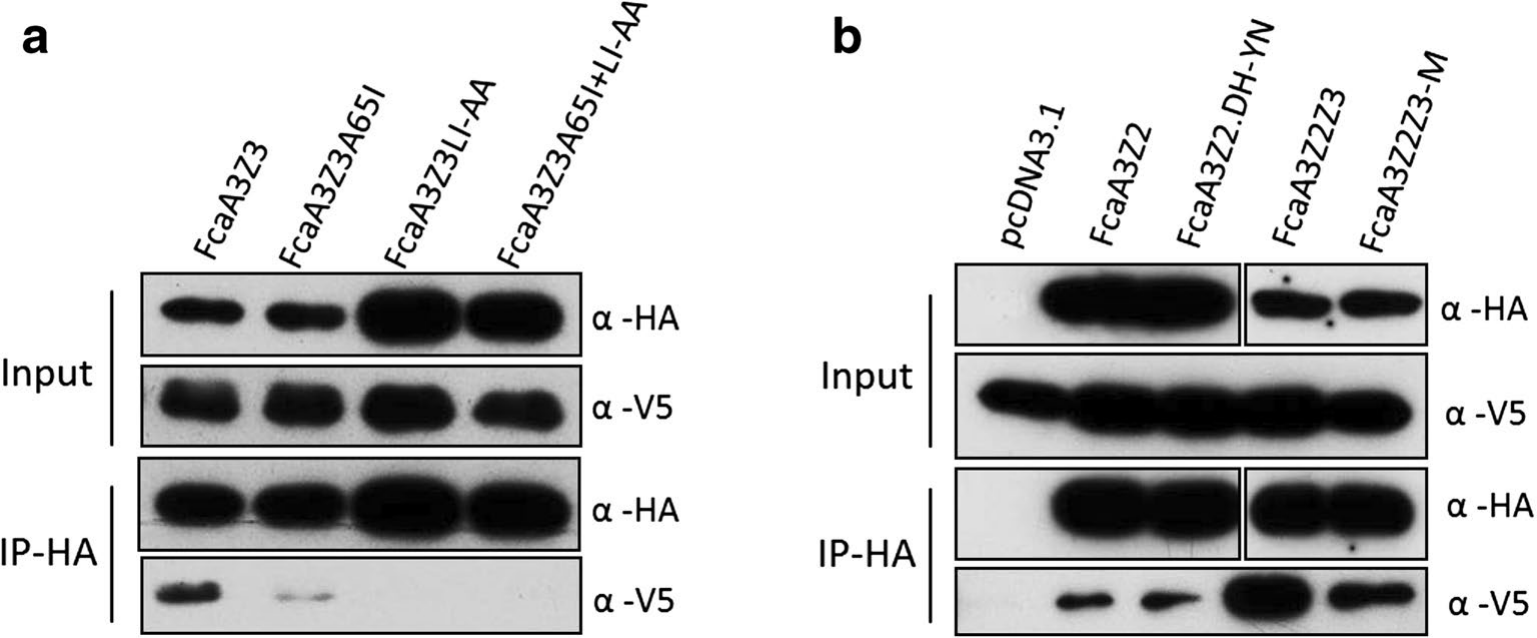
*Differential binding of FIV Vif to wild*-*type and mutant feline A3*s. **a** Expression plasmids for FcaA3Z3s wild-type and mutants (all with HA-tag) and FIV Vif-TLQAAA (V5 tag) were co-transfected into 293T cells. The proteins were immunoprecipitated by α-HA beads and analyzed by immunoblots using anti-HA and anti-V5 antibodies. **b** Expression plasmids for FcaA3s (FcaA3Z2, FcaA3Z2.DH-YN, FcaA3Z2Z3 and FcaA3Z2Z3-M, all with HA-tag) and FIV Vif-TLQAAA (V5-tag) and were co-transfected into 293T cells, pcDNA3.1 (+) served as an A3-free control. 48 h later, cells were harvested, proteins were immunoprecipitated by α-HA beads. The FcaA3s and FIV Vif proteins were detected by anti-HA and anti-V5 antibodies, respectively

To check whether the FIV Vif-resistant mutant A3s displayed modified binding to Vif, wild-type and mutated A3s together with FIV Vif-TLQAAA were co-expressed and analyzed by anti-HA immuno-precipitation (Fig. 6). Wild-type cat A3Z3 precipitated FIV Vif (Fig. 6a), consistent with a direct interaction of both proteins (Fig. 1c). Only very little Vif bound to A3Z3.A65I and no Vif was detected in precipitations of A3Z3.LI-AA and A3Z3.A65I + LI-AA (Fig. 6a). However, when we examined wild-type A3Z2 and the DH-YN mutant, we detected similar amounts of Vif in both precipitations. Wild-type A3Z2Z3 bound high levels of Vif, and this binding was much reduced by the mutated variant A3Z2Z3-M (Fig. 6b). Globally, our observations suggest that A65I and LI-AA mutations in A3Z3 abolished FIV Vif binding, while hitherto not identified residues mediate Vif binding in A3Z2.

### Structural analysis of feline A3Z2Z3

**Fig. 7:**
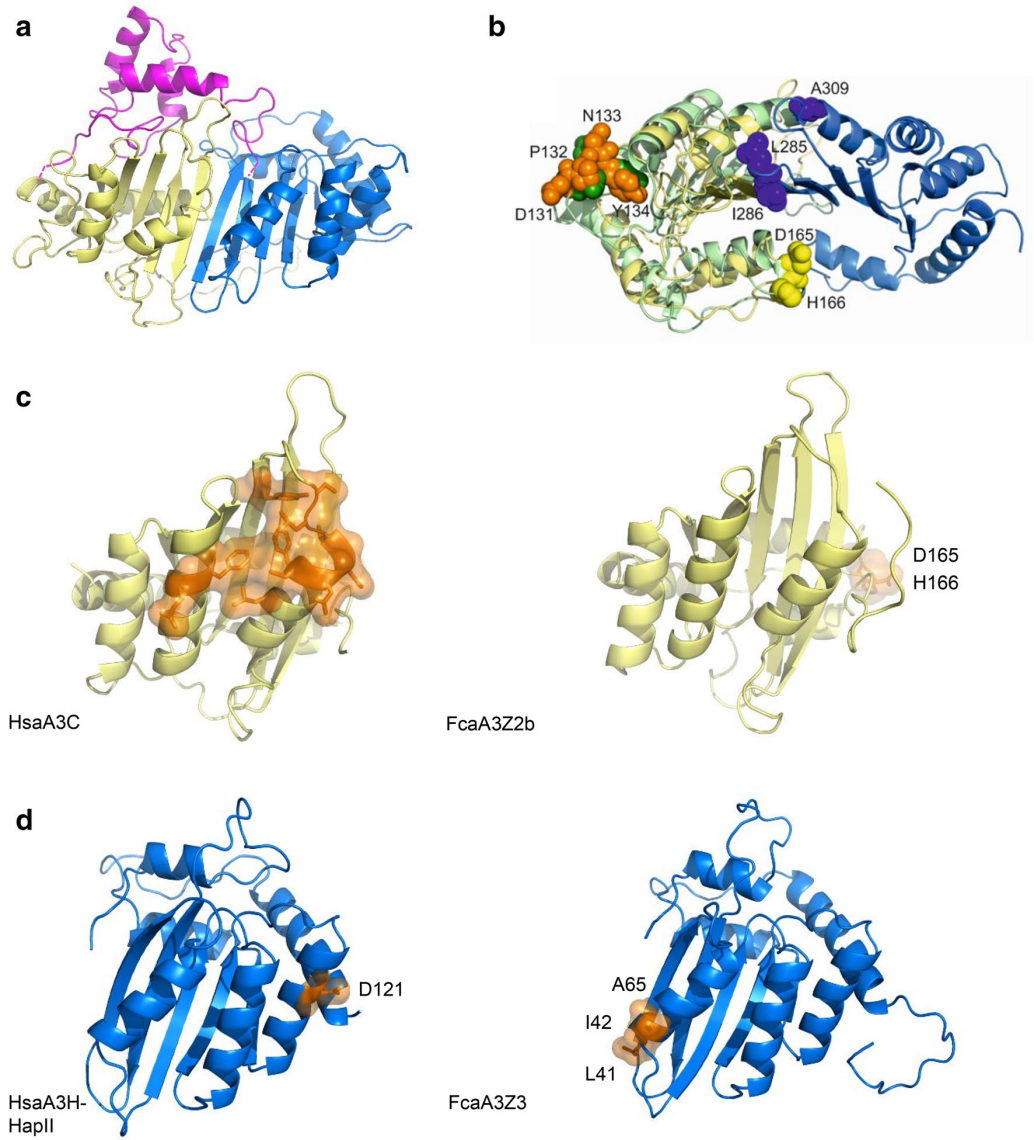
Composite model of feline APOBEC3 and locations of residues mediating Vif binding. **a** Structural model of FcaA3Z2Z3 including the linker (*pink*) at a putative location above the Z2 (*yellow*) and Z3 (*blue*) domains; the linker connections to the Z2 and Z3 domains are highlighted by *dashed lines*. [69]. **b** Structural model of FcaA3Z2Z3 rotated by 90° with respect to **a** (Z2 domain: *yellow*; Z3 domain: *blue*; the linker region and parts of the N-terminus of Z2 and the C-terminus of Z3 for which no structure could be modeled were omitted for clarity). Residues in sphere representation in *yellow* (D165/H166), those in *blue* (L285/I286/A309), and those in *orange* (D131-Y134). Residues sequentially equivalent to the latter in the soluble N-terminal Vif-binding domain (sNTD) of A3G (PDB ID 2MZZ; *pale green*) are colored in green; these residues are part of the Vif-binding regions of the sNTD [68]. **c** Crystal structure of human A3C (PDB ID: 3VOW) and structural model of feline A3Z2b depicting the positions of respective HIV-1 Vif and FIV Vif binding sites. The domains are orientated as the Z2 domain in **a**. **d** Structural model of human A3H-HapII and feline A3Z3 depicting the positions of respective HIV-1 Vif and FIV Vif binding sites. The domains are orientated as the Z3 domain in **a**. Key residues involved in Vif binding are labelled (except human A3C), represented in sticks and highlighted with its surface in *orange color*

To identify the position of residues in feline A3s that, when mutated, prevent binding of FIV Vif and A3Z2Z3 degradation, a structural model of feline A3Z2Z3 was generated, initially aligning its sequence to the human full-length A3G model [58]. Surprisingly, the alignment indicated a large insertion in the Z2–Z3 linker region in the feline sequence that is not present in the human counterpart (Additional file 1: Fig. S5). This domain insertion spans 46 residues, extending the feline A3Z2Z3 linker to 83 residues compared to 27 residues in humans. The structure of the 83-residue linker was predicted using TopModel [59, 60]. Although the five identified template structures show only a low sequence identity with respect to the linker (up to 19.4 %; see “Methods” section), they all share a homeo-box domain fold [61]. The best three templates were aligned to the linker sequence (Additional file 1: Fig. S5) and used for structure prediction. The rest of the feline A3Z2Z3 protein was predicted using the homology model of human A3G [58] as a template. Finally, the linker domain and the rest of the feline A3Z2Z3 protein were manually docked, sequentially connected, and unstructured parts of the linker domain were energy minimized (Fig. 7a). While this cannot be expected to result in an exact structural model, it provides a representation where the linker domain insertion could be located with respect to the rest of the feline A3Z2Z3 protein.

The five residues in feline A3s that, when mutated, prevent binding of FIV Vif and A3Z2Z3 degradation (D165, H166, L285, I286, A309; the last three corresponding to L41, I42 and A65 of A3Z3), are located opposite to the putative location of the linker domain and are at the boundary between the Z2 and Z3 domains (Fig. 7b). The predicted HIV-1 Vif binding regions in human A3G, A3C and A3H are additionally depicted in Fig. 7b–d, respectively. For A3C and A3H, the predicted HIV-1 Vif binding regions are spatially clearly separated from the respective five residues identified here in feline A3s (Fig. 7c, d). One may thus speculate that our findings indicate a FIV Vif binding region in feline A3 different from the ones described for HIV-1 Vif in human A3s.

### FIV Vif-resistant feline A3s are antiviral

**Fig. 8:**
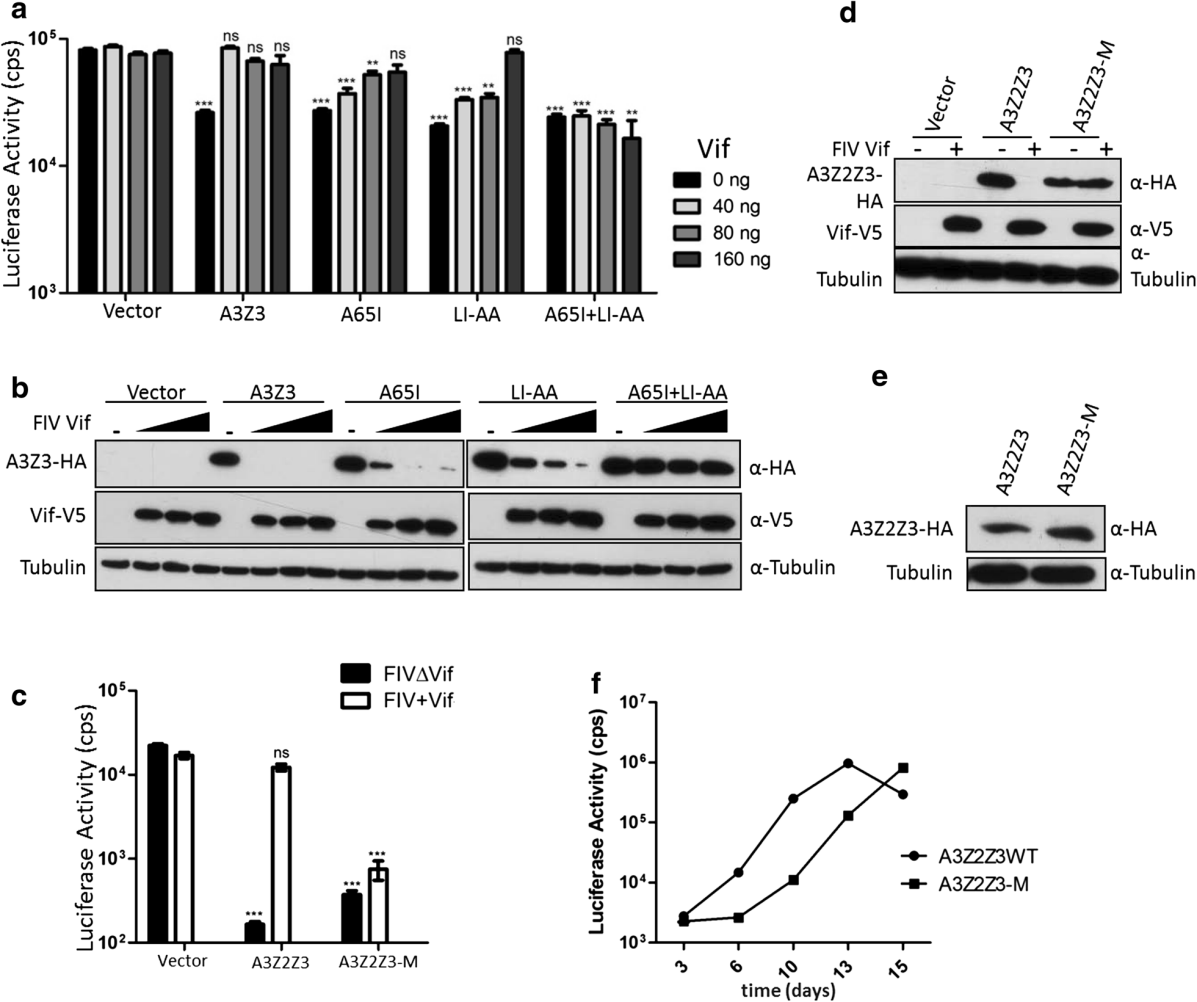
The impact of mutations in FcaA3s on FIV infectivity. **a** FIVΔ*vif* luciferase reporter virions were produced in the presence of feline A3 expression plasmids (FcaA3Z3 wild-type, FcaA3Z3.A65I, FcaA3Z3.LI-AA or FcaA3Z3.A65I + LI-AA, 250 ng plasmid) with increasing amounts of FIV Vif (0, 40, 80 and 160 ng plasmid), pcDNA3.1 (+) was added as a control (vector). Infectivity of reporter vectors was determined by quantification of luciferase activity in 293T cells transduced with vector particles. **b** Cell lysates of FIV producer cells examined in **a** were used to detect the expression of FcaA3Z3 and FIV Vif by anti-HA and anti-V5 antibodies, respectively. Cell lysates were also analyzed for equal amounts of total proteins using anti-tubulin antibody. **c** The impact of FcaA3Z2Z3 and FcaA3Z2Z3-M on infectivity of FIVΔ*vif* luciferase reporter vectors in the presence of FIV Vif. 293T cells were co-transfected FIVΔ*vif* luciferase reporter viruses with 1000 ng FcaA3s and 100 ng FIV Vif expression plasmids. 48 h later, FIV particles were used to infect 293T cells, and infectivity was determined by quantification of luciferase activity. **d** Cell lysates for FIV producer cells used in **c** were analyzed by immunoblots to detect the expression of FcaA3Z3s and FIV Vif by anti-HA and anti-V5 antibodies, respectively. Cell lysates were also analyzed using anti-tubulin antibody. **e**, **f** Spreading replication of HIV-1 expressing FIV Vif (NL-Bal.*vif*_FIV_) is inhibited by feline A3Z2Z3-M. **e** Immunoblot analysis of HOS.CD4.CCR5 cells stable expressing feline A3Z2Z3 proteins. A3 proteins were detected by anti-HA antibody. Anti-tubulin served to demonstrate equal protein loading. **f** HOS.CD4.CCR5 cells expressing either wild-type A3Z2Z3 or A3Z2Z3-M were infected by NL-Bal.*vif*_FIV_ with an MOI of 0.01 and virus replication was monitored by using the cell culture supernatant for infection of TZM-bl luciferase reporter cells. *Asterisks* represent statistically significant differences: ***p < 0.001; **0.001 < *p* < 0.01; *0.01 < *p* < 0.05; ns, *p* > 0.05 (Dunnett *t* test)

In the next set of experiments, we investigated whether feline A3s carrying the putative Vif-binding mutations displayed antiviral activity and resistance against Vif in FIV infections. We generated FIV luciferase reporter viruses by co-expression with either no A3 or with A3Z3, A3Z3.A65I, A3Z3.LI-AA or A3Z3.A65I + LI-AA and increasing levels of the FIV Vif plasmid (0–160 ng). Vector particles were normalized for reverse transcription (RT) activity, and luciferase activity was quantified 2 days post infection (Fig. 8a). All feline A3Z3s, either wild-type or mutants, were able to inhibit to the same extent Vif-deficient FIV, demonstrating that the described mutations do not hinder the potential for antiviral activity. Wild-type feline A3Z3 was fully counteracted by the lowest amount of Vif plasmid (40 ng) (Fig. 8a), matching well complete degradation observed in the lysates of FIV-producing cells (Fig. 8b). Opposite to the homogenous behavior in the absence of Vif, mutated A3Z3s showed variable resistance to Vif-counteraction, as was obvious in the levels of remaining A3 signal in the cell lysates of the FIV-producing cells (Fig. 8b). Intermediate amounts of *vif*-encoding plasmid (40–80 ng) partially counteracted the inhibition of A3Z3.A65I or A3Z3.LI-AA mutants, and higher levels of Vif (160 ng plasmid) recovered infectivity of FIVs produced in the presence of A3Z3.A65I and A3Z3.LI-AA. However, even the highest levels of Vif were not able to counteract the antiviral activity of A3Z3.A65I + LI-AA (Fig. 8a, b). The importance of Z2- and Z3-mutations in feline A3Z2Z3-M was characterized with 100 ng of FIV Vif plasmid. FIV luciferase viruses were produced and examined as described above using A3Z2Z3 and A3Z2Z3-M. FIV Vif restored the infectivity to levels similar to those in the absence of A3Z2Z3, while A3Z2Z3-M strongly inhibited FIV, either with or without Vif expression (Fig. 8c). The immunoblots of the corresponding FIV producing cells confirmed protein expression and Vif-dependent degradation of the wild-type A3Z2Z3 protein (Fig. 8d). To explore whether stable expression of A3Z2Z3-M can impact spreading infection, human HOS.CD4.CCR5 cells expressing either wild-type or mutated A3Z2Z3 were established (Fig. 8e, Additional file 1: Fig. S6) and infected by HIV-1 expressing FIV Vif (HIV-1*vif*_FIV_) [36]. FIV could not be investigated directly, because there are no feline cell lines known to be negative for A3 expression, and FIV cannot replicate in human cell lines. Whereas HIV-1*vif*_FIV_ was detected at day six in the supernatant cells with wild-type A3Z2Z3, HOS cells expressing the A3Z2Z3-M showed a much delayed kinetic of viral replication (Fig. 8f). This observation suggests that the engineered A3Z2Z3-M protein also gained the capacity to restrict FIV Vif during multi-rounds of replication.

Encapsidation of A3 proteins in nascent virions is required for their antiviral activity. We investigated first whether mutated feline A3s could be differentially encapsidated into nascent virions. For this, we produced Vif-deficient FIV particles during expression of the different A3 proteins, measured the differential infectivity of the particles (Additional file 1: Fig. S7A) and subjected virus lysates to immunoblot analysis (Additional file 1: Fig. S7B). Results showed that wild-type and mutated feline A3s were detected in the concentrated FIVs (VLPs). However, while wild-type A3Z3 was less efficiently packaged compared with the A3Z3.A65I + LI-AA mutant, the wild-type A3Z2Z3 was detected in virions in higher abundance than the A3Z2Z3-M variant (Additional file 1: Fig. S7B). We addressed then the question whether encapsidated mutated feline A3s effectively exerted their cytidine deaminase activity onto the FIV genome in the virion. To tackle this question, cells were infected with FIV produced during cellular expression of feline A3Z3, A3Z3.A65I + LI-AA, A3Z2Z3 or A3Z2Z3-M, in the absence of A3 expression as a negative control, or during expression of human A3G as positive control. Total DNA was isolated from infected cells and subjected to differential DNA denaturing PCR (3D-PCR) [62] on the viral vector encoded luciferase gene 12 h post infection. Based on the overall nucleotide content, 3D-PCR amplifies PCR products at different denaturing temperatures (Td), with amplicons with higher A + T content displaying lower denaturing temperatures than amplicons with higher G + C content. The net effect of the cytidine deaminase A3 activity is thus expected to lower the denaturing temperature of the target DNA, leading to lower Tds values. Indeed, FIV virions produced in the absence of A3s yielded 3D-PCR products with the lowest Td of 86.3 °C, whereas all FIV virions produced during A3 expression resulted in 3D-PCR products with decreased Tds (as low as 84.2 °C) (Additional file 1: Fig. S7C). This indicates that the wild-type and mutant feline A3s display enzymatic deamination activities.

### The linker in feline A3Z2Z3 is targeted by HIV-2 and SIVmac/smm Vifs

**Fig. 9:**
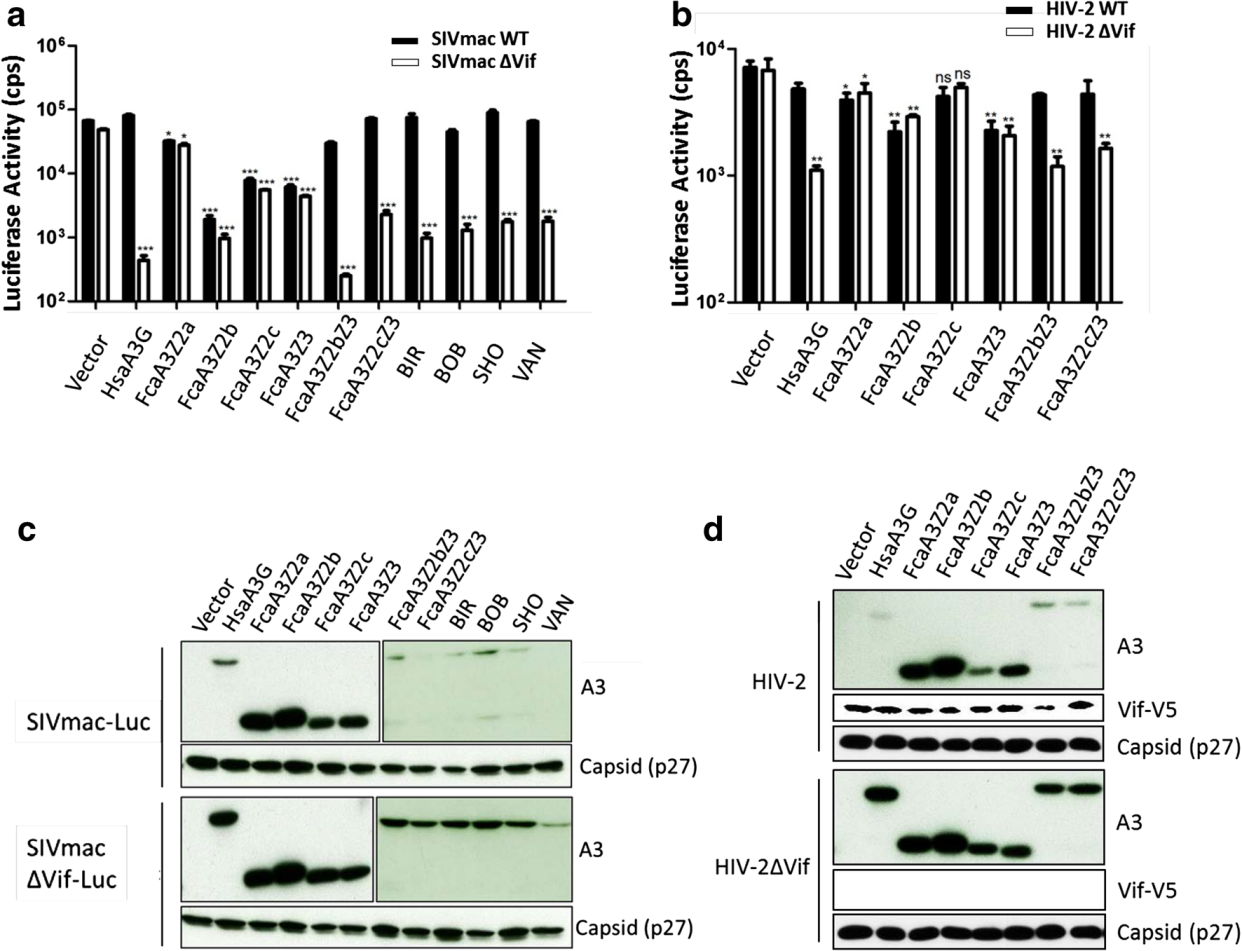
*SIVmac and HIV*-*2 escape inhibition by Fca*A3*Z2Z3.* **a**, **b** 293T cells were transfected with expression plasmids for **a** SIVmacΔ*vif*-Luc (SIVmacΔ*vif*) or SIVmac-Luc (SIVmac WT) or **b** HIV-2Δ*vif*-Luc (HIV-2Δ*vif)* or HIV-2Δ*vif*-Luc + HIV-2 Vif (HIV-2 WT), together with expression plasmids for HsaA3G or FcaA3s, pcDNA3.1 (+) was used as a control (vector). Reporter virus infectivity was determined by quantification of luciferase activity in 293T cells transduced with vector particles after normalizing for reverse transcriptase activity. *Luc* luciferase. **c** Lysates of SIVmac producer cells were used to detect the expression of FcaA3s and SIVmac capsid by anti-HA and anti-p27 antibodies, respectively. SIVmac Vif cannot be detected because of the unavailability of a suitable antibody. **d** Lysates of HIV-2 producer cells were used to detect the expression of FcaA3s and HIV-2 Vif by anti-HA and anti-V5 antibody, respectively. BIR, BOB, SHO and VAN represent FcaA3Z2Z3s including polymorphic sequences of exon 4 of four different *Felis catus* breeding lines: *BIR* Birman, *BOB* Japanese Bobtail, *SHO* British Shorthair, *VAN* Turkish Van. *Asterisks* represent statistically significant differences: ***p < 0.001; **0.001 < *p* < 0.01; *0.01 < *p* < 0.05; ^ns^ *p* > 0.05 (Dunnett *t* test)

We and others have observed that SIVmac Vif can induce degradation of feline A3Z2Z3 (Fig. 1b) [46, 51]. Figure 1b demonstrates that the Vifs of SIVsmm and HIV-2 also display this phenotype and are able to degrade feline A3Z2Z3. To elucidate this unexpected capacity of primate lentiviruses to counteract feline A3s in the context of viral infections, we generated luciferase reporter viruses for SIVmac and HIV-2 (Fig. 9). SIVmac or SIVmacΔ*vif* luciferase reporter viruses [63] were produced in the absence or presence of human A3G, feline A3Z2a, A3Z2b, A3Z2c, A3Z2bZ3, A3Z2cZ3 or A3Z2bZ3s that included polymorphic residues found in exon 4 of different *F. catus* breeding lines (Birman, Japanese Bobtail, British Shorthair, Turkish Van [36]). The Vif proficient virus SIVmac-Luc expresses Vif in its natural expression context; however Vif lacks a tag for detection. Viral particles were normalized for RT activity and luciferase activity of infected cells was quantified 2 days post infection (Fig. 9a). We found that double-domain feline A3s strongly inhibited Vif-deficient SIVmac, and that Vif expression fully counteracted this antiviral activity, showing therefore a similar pattern to human A3G. However, Vif expression did not affect inhibition of SIVmac by single domain A3s (Fig. 9a). The corresponding immunoblots of the virus producing cells showed Vif-dependent degradation of human A3G and of all feline A3Z2Z3s inspected. Feline A3Z2s and A3Z3 displayed a resistance to degradation by Vif proficient SIVmac (Fig. 9c). We performed a similar experiment using a HIV-2 luciferase reporter virus [64], which is a three-plasmid lentiviral vector system that requires Vif to be co-expressed from a separate plasmid (Fig. 9b). Using this system, we found that HIV-2 Vif counteracted the antiviral activity of human A3G, feline A3Z2bZ2 and of A3Z2cZ3. Again, the antiviral activity of feline single-domain A3s could not be inhibited by HIV-2 Vif (Fig. 9b). The immunoblots of the virus producing cells showed a Vif-dependent depletion of human A3G as well as of the feline double-domain A3s (Fig. 9d).

**Fig. 10:**
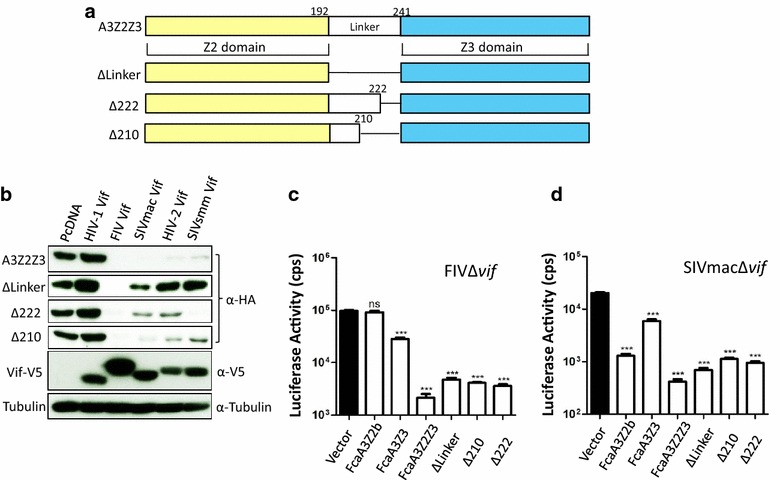
The linker region in FcaA3Z2Z3 is important for HIV-2/SIVmac/smm Vif induced degradation. **a** Schematic representation of FcaA3Z2Z3 mutants (ΔLinker, Δ222, Δ210). Z2 and Z3 domains are shown as *yellow* or *blue rectangle*, respectively. The linker from amino acid 192–241 is shown as *white rectangle*. **b** The Vif sensitivity of FcaA3Z2Z3 mutants was analyzed by co-transfecting expression plasmids of the A3 mutants together with lentiviral Vifs (from HIV-1, FIV, SIVmac, HIV-2 or SIVsmm) into 293T cells. The expression of FcaA3s and Vifs were confirmed by immunoblots and anti-HA and anti-V5 antibodies, respectively. Cell lysates were also analyzed for equal amounts of total proteins using anti-tubulin antibody. **c** Test of antiviral activity of linker mutants against FIVΔ*vif*-luciferase and **d** against SIVmacΔ*vif*-luciferase. Infectivity of virions generated with wild-type and mutated A3 was measured on 293T cells transduced by RT activity normalized particles. *CPS* counts per second. *Asterisks* represent statistically significant differences: ***p < 0.001; **0.001 < *p* < 0.01; *0.01 < *p* < 0.05; ^ns^ *p* > 0.05 (Dunnett *t* test)

The Vif-mediated degradation profile exclusive to A3Z2Z3s may indicate that the HIV-2/SIVmac/smm Vifs require for interaction with the feline A3Z2Z3 a protein domain that is absent in the single-domain A3Z2 or A3Z3. We speculated that the homeo-box domain insertion (linker region) could play a central role in these Vif interactions. To test our hypothesis, three constructs were assayed: an A3Z2Z3 in which the linker was deleted (ΔLinker); and two versions of A3Z2Z3 in which either residues 223–240 (Δ222) or residues 211–240 (Δ210) in the linker were removed (Fig. 10a). All these constructs successfully expressed protein upon transfection, and FIV Vif was able to degrade all of them. Only the linker truncations Δ222 and Δ210 were efficiently degraded by Vif of HIV-2/SIVmac/smm, whereas the ΔLinker construct showed very little degradation (Fig. 10b). We extended this experiment and analyzed the degradation with increasing levels (0, 20, 50, 150 or 250 ng) of SIVmac or HIV-2 Vif expression plasmid (Additional file 1: Fig. S8). Interestingly, the A3Z2Z3 lacking the linker domain (ΔLinker) showed dose-dependent moderate degradation, while mutants Δ222 and Δ210 showed a HIV-2/SIV Vif-dependent degradation similar as the wildtype A3Z2Z3 protein (Fig. 10b, Additional file 1: Fig. S8). To characterize the linker mutant A3s for functional antiviral activity, FIVΔ*vif* and SIVmacΔ*vif* luciferase reporter viruses were generated in the presence of wild-type and mutated A3s (Fig. 10c, d, Additional file 1: Fig. S9). Immunoblots of the viral particles showed that all A3s were encapsidated (Additional file 1: Fig. S9). Consistently in both viral systems, A3Z2Z3 moderately lost antiviral activity when part or the complete linker was deleted (ΔLinker, Δ210, Δ222) (Fig. 10c, d). Together, our results suggest that the linker domain enhances the antiviral activity of feline A3Z2Z3 but is not essentially required for it and that the linker is important for HIV-2/SIVmac/smm Vif degradation of feline A3Z2Z3. Whether the linker domain forms part of the HIV-2/SIV Vif interaction surface will be an important future question.

Because the linker insertion is absent in human A3s, we tried to learn more about the evolution of this unique domain. The DNA sequences in A3Z3 exon 2 encoding for the linker region in the double-domain A3Z2Z3 proteins are extremely conserved among members of Felinae and Pantherinae. The linker sequence is indeed more conserved than the corresponding Z2 and Z3 domains, the evolutionary distances being 0.044 ± 0.006 for the Z2 stretch, 0.011 ± 0.006 for the linker and 0.018 ± 0.004 for the Z3 stretch (overall average pairwise nucleotide distance ± bootstrap standard error estimate). The evolutionary origin of the linker remains nevertheless obscure, as systematic BLASTn and BLAT searches using this linker sequence as seed did not retrieve hits beyond spurious matches. However, tBLASTn successfully retrieved hits associated with A3 genes: in the 5′ untranslated region of the A3 gene (XR_434780) in the Weddell seal genome, in the 5′ untranslated region of the A3 gene (FJ716808) in the camel genome, as well as in the 5′ regulatory region of the A3 gene (FJ716803) in the pig genome. The only very remote hit in primates with linker-similar sequence could be located in an A3 gene of tarsier (XM_008049574.1), but similarity levels do not allow in this case claiming common ancestry.

### HIV-1 Vif weakly interacts with feline A3Z2Z3

The finding that HIV-2 Vif counteracts one of the feline A3s reinforces the view that the initially described species-specificity of Vifs [63] is not absolute [36, 52]. For the generation of an HIV-1 animal model based on the cat, it would of advantage to understand whether feline A3 proteins are structurally accessible for HIV-1 Vif. We show here that HIV-1 fails to degrade feline A3 proteins (Figs. 1b, 10b) and appears only to bind weakly to the feline A3Z2Z3 protein compared to FIV Vif (Additional file 1: Fig. S10A).

The structural model of feline A3Z2Z3 was used to rationalize the binding of HIV-1 Vif to A3Z2Z3. When comparing the amino acid sequences of A3G and feline A3s, we noticed that the HIV-1 Vif binding domain 124-YYFWDPDY-131 is conserved in feline A3Z2 (Additional file 1: Fig. S10B). This domain spans amino acid residues with a well-characterized role in Vif-binding, such as 128-DPD-130 [65] and the recently characterized Y125 [66] in the β4-α4 loop of human A3G [67]. In the feline A3Z2 domain we find DPN instead of the DPD motif; however, in human A3G DPN binds to HIV-1 Vif as wild-type DPD [65]. As our structural model of A3Z2Z3 in comparison to the soluble N-terminal domain (sNTD) of A3G [68] revealed that the two regions around these residues are similarly accessible (Fig. 7b), we attempted to restore binding of HIV 1 Vif to feline A3Z2Z3 by a N133D mutation, resulting in a YYFWDPD133Y motif sequentially identical to the one in A3G (Additional file 1: Fig. S10b). We did not observe degradation of A3Z2Z3.N133D by HIV-1 Vif (Additional file 1: Fig. S10C), however; neither were mutations of P132 to introduce additional side chain interactions successful in that respect (Additional file 1: Figs. S10B, S10C). As to a possible explanation, for A3C, which is structurally highly similar to the sNTD of A3G [68], another motif of residues critical for Vif binding was found, centering on F75, Y86, F107, and H111 [69] (Fig. 7c). The sequentially equivalent residues of A3Z2Z3 are F78, Y89, F110, and Y114 such that the exchange of His versus Tyr may explain the failing of the binding of HIV-1 Vif. Another possible explanation for A3Z2Z3 is given by the occlusion of space required for HIV-1 Vif binding due to the presence of the predicted linker domain, where the long unstructured regions at the beginning and the end of the structured linker part may make it possible that the linker domain tips over the Z2 domain, that way shielding the putative HIV-1 Vif binding region (Fig. 7a, b).

## Discussion

The A3 restriction factors are of extraordinary importance for the evolution and pathogenicity of lentiviruses and likely also of most other retroviruses. Here we identified A3 residues that are relevant for the FIV Vif interaction with both single-domain A3s, A3Z2 and A3Z3 (results are summarized in Table 1). In addition, we analyzed a unique A3 protein insertion domain called linker present in the feline A3Z2Z3 protein. The linker is suggested to form a homeo-box domain and mediates the sensitivity of A3Z2Z3 to degradation by Vifs of the HIV-2/SIVmac/smm group of primate lentiviruses.

**Table 1:** Summary Vif-mediated A3 degradation

Feline A3^a^	Degradation by Vif				Rescue of infection by Vif^b^		
	FIV	HIV-1	HIV-2	SIVmac/smm	FIV	HIV-2	SIVmac
A3Z2	++	−	−	−	ND	−	−
A3Z2.DH-YN	−	ND	ND	ND	ND	ND	ND
A3Z3	++	−	−	−	++	−	−
A3Z3.A65I	±	ND	ND	ND	±	ND	ND
A3Z3.LI-AA	±	ND	ND	ND	±	ND	ND
A3Z3.A65I + LI-AA	−	ND	ND	ND	−	ND	ND
A3Z2Z3	++	−	++	++	++	++	++
A3Z2Z3-M	−	ND	++	++	−	ND	ND
A3Z2Z3ΔLinker	++	−	±	±	ND	ND	ND

Degradation of A3 by Vif: ++, mostly degraded; ±, partial degradation with high amount of Vif; −, no degradation

Rescue of infection by Vif: ++, complete rescue; ±, partial rescue; −, no rescue

*ND* not done

^a^Feline A3: A3s from domestic cat *Felis catus*

^b^Experiments to rescue the infection were not done with HIV-1 and SIVsmm

Our knowledge about the interaction regions of A3s and of human and non-human lentivirus Vifs is limited. It was discussed that Vif is not simply a linker between the substrate A3 and the E3 ubiquitin ligase [70, 71]. In our study we investigated the interaction of three groups of Vif proteins (FIV, HIV-2/SIV, HIV-1) with feline A3s. Previous experimental evidence described residue A65 in feline A3Z3 in modulating the sensitivity to FIV Vif [56]. We identified here two additional residues (L41, I42) in feline A3Z3 whose combined mutation resulted in an A3 protein that was resistant even to degradation by very high amounts of co-expressed FIV Vif. The mutated feline A3Z3 protein clearly showed reduced binding to FIV Vif, supporting the model that Vif binding to A3 is needed for A3 degradation. In feline A3Z2 residues D165 and H166 were also found to regulate the FIV Vif induced degradation, but mutations in these positions did not block the binding to FIV Vif in co-immunoprecipitation assays. This observation demonstrates that Vif binding to A3s is not sufficient for A3 degradation. Supporting evidence that Vif interaction is necessary but not sufficient is coming from reports describing that HIV-1 NL4-3 Vif binds A3C mutants, A3B and A3H without inducing APOBEC3 degradation [71–73]. The qualitative co-immunoprecipitation assays used in our study did not much differentiate the binding strength of individual Vif-A3 pairs, and it is very well possible that a weak interaction of e.g. HIV-1 Vif with feline A3Z2Z3 is below a threshold to form a stable E3 ligase complex. However, the binding of mutated feline A3Z2.DH-YN to FIV Vif appeared to be robust, indicating a more complex mechanism. Studies on HIV-1 Vif binding to human A3B and A3H similarly concluded that the interaction strength is not the only determinant for complete Vif-mediated degradation, and the individual interfaces of the A3-Vif pair additionally regulate degradation [72].

Recently, Richards et al. [74] presented a wobble model of the evolution of the Vif-A3 interaction. This model implicates that Vif forms several interactions, of which some are essential and some provide additional stabilizing contacts. Based on this idea, only if Vif forms a sufficient network of interactions with its A3 binding partner, a functional interaction is made. Suboptimal, destabilized interactions could be restored by the evolution of compensatory changes in Vif–A3 interface. It is thus possible that in feline A3Z3 residue A65 and L41, I42 are major independent interactions in the Vif-A3 interface, whereas in feline A3Z2 D165 and H166 represent one of the relevant interacting points for FIV Vif complex formation, while additional contact points still exist. Such a suboptimal Vif-A3 interaction might, for example, not be sufficient to facilitate E3 ligase conjugation of K48-linked polyubiquitin chains that are generally recognized by the proteasome.

The exact Vif-A3 interfaces are not known, because high-resolution structures have been only solved of single proteins such as of Z1- and Z2-domain human proteins (A3A, A3C), of the N-terminal Z2- and C-Terminal Z1-domain of human A3G, of the C-terminal Z2-domain of A3F and of HIV-1 Vif [67, 75, 76]. The structures of the full-length double domain A3s are unknown, however. Human A3Z1s and A3Z2s are globular proteins with six α-helices and five β-sheets arranged in a characteristic motif (α1-β1-β2/2′-α2-β3-α3-β4-α4-β5-α5-α6) [67, 76]. In human A3C, A3D and A3F, the HIV-1 Vif binding site is conserved and located in a hydrophobic cavity and on the surrounding surface of the α2, α3 and α4 helices [69, 77, 78]. In human A3G, HIV-1 Vif binds a surface different to the binding region in A3C/D/F, with residues Y125, 128-DPN-130 in the β4-α4 loop being important for HIV-1 Vif binding [65, 66]. In the human Z3 protein A3H, binding of HIV-1 Vif is mediated by residue 121 (either E or D) [79, 80]. Based on our structural model of feline A3Z2Z3 (Fig. 7b), we locate the residues important for FIV Vif binding in feline A3Z2Z3 at the domain boundary of the Z2 and the Z3 domains, distant to the binding motifs in human A3s (Fig. 7c, d).

In feline A3Z2, the presumed HIV-1 Vif-binding domain of human A3G, the β4-α4 loop, is conserved. Nevertheless, HIV-1 Vif fails to degrade feline A3Z2 or A3Z2Z3 despite the presence of the well-characterized residues DPN (in A3G residues 128–130) and Y125 [65, 66]. Based on our structural model, we suggest that the β4-α4 loop of feline A3Z2 is surface exposed. This suggests that the Z2-domain of human A3G contains in addition to the Y125, 128-DPN-130 motif residues for HIV-1 Vif binding that are absent or hidden in feline A3Z2 or A3Z2Z3. Indeed, the presences of such important residues outside this motif in A3G were recently postulated [68, 81]. In addition to FIV Vif, we and others found previously that HIV-2/SIVmac/smm Vifs induce degradation of feline A3Z2Z3 [46, 51] by possibly targeting the unique linker domain. The previously called linker, a domain insertion in feline A3Z2Z3, is not found in any double-domain A3 protein of human or mouse origin. Our modelling results suggest that the insertion forms a homeo-box domain-like structure that protrudes the Z2-Z3 structure.

In general, it appears that double-domain A3 proteins display stronger antiviral activities than single-domain A3s. The evolution of double-domain encoding A3 genes could thus have been most likely adaptive, as it significantly increased the host fitness against retroviral infections. Our results suggest that primates and felids could have evolved double-domain A3s through different routes. The sequence of the linker insertion is located in 5′UTR of the felid A3Z3 gene in exon 2, which is exclusively translated in read-through transcripts spanning the A3Z2 and A3Z3 genes in felines (Fig. 1a). The sequence encoding for exon 2 seems to be restricted to members of Felinae and Pantherinae. In this sense, the A3Z2Z3 linker region resembles an orphan domain specific to Feliformia, and the linker could thus be a synapomorphy of this clade. Nevertheless, homology searches identified an enrichment of significantly remote tBLASTn hits associated with regulatory or non-coding regions of A3 genes in the genomes of different species, in the carnivore Weddell seal and in the artyodactyls pig and camel. This concentration of sequences with a possible common origin with the feline A3Z2Z3 linker found in the close vicinity of the A3 genes in other species within Laurasiatheria suggests that the linker could have been recruited as a coding sequence into the feline A3Z2Z3 mature mRNA from a pre-existent non-coding possibly regulatory sequence, in an example of gain of function. This sequence could have been recruited after point mutation/s resulting in stop codon removal, introduction of frameshifts or unmasking previously cryptic functional sites [82] during the evolution of carnivores, after the split Caniformia/Feliformia but before the split Pantherinae/Felinae. In the case of primates and of rodents there are no descriptions of read-through transcripts of single domain A3s resulting in mRNAs encoding double-domain A3s. Instead, the human heterologous double domain A3s (i.e. A3B and A3G, both being A3Z2Z1) or homologous double domain A3s (i.e. A3D and A3F, both being A3Z2Z2) could have evolved after the fusion of head-to-tail duplicated genes, as the several rounds of gene duplication in the evolutionary history of the A3 locus in primates suggest [3].

During our evolutionary analysis of the A3Z3 genes, we found here for the first time duplicated A3Z3 genes. A3Z3 duplications were identified in the genomes of different carnivores (the giant panda, the polar bear, the Weddell seal and the walrus), but were not found in dog and ferret and also not in any felid. The most parsimonious hypothesis would be that a duplication event occurred within Caniformia, after the basal split of Canidae. However, given the inferred position of the most recent common ancestor of all A3Z3 in carnivores, and given the within-clades and between-clades evolutionary distances (Additional file 1: Fig. S3), we propose that an ancient A3Z3 duplication event may have occurred prior to the Caniformia/Feliformia split. One of the in-paralogs would have disappeared in the Felidae ancestor, and at least in the dog genome, while both copies would have been maintained in most lineages within Caniformia (the absence in the ferret genome should be confirmed when better quality data are available).

## Conclusions

Host-virus arms races formed the Vif-A3 interactions. Our data support that the evolution of HIV-1, HIV-2 and FIV follow intrinsic currently unexplained evolutionary pathways adapting to the antiviral A3 repertoire. This study also revealed that the A3 gene evolution included newly identified duplications (in-paralogs) of A3Z3 genes in some caniformia and the inclusion of a homeobox-domain in the feline A3Z2Z3 protein. This homeobox domain insertion may reflect a transitional situation (read-through transcription) of the evolutionary development of double Z-domain containing A3 proteins. Further resolution of the interaction surface of feline A3s with Vif proteins will help us to understand the biochemistry of these interactions and may give us tools to explore the HIV-1 Vif interaction with human A3s.

## Methods

### Cells and transfections

HEK293T (293T, ATCC CRL-3216), HOS (ATCC CRL-1543) and TZM-bl cells (NIH AIDS Reagent program [83, 84]) were maintained in Dulbecco’s high-glucose modified Eagle’s medium (DMEM, Biochrom, Berlin, Germany) supplemented with 10 % fetal bovine serum (FBS), 2 mM l-glutamine, penicillin (100 U/ml), and streptomycin (100 μg/ml). Stable A3 expressing cells: FcaA3Z2Z3 wild type and mutant pcDNA-constructs were digested by BglII, and then were transfected into HOS.CD4.CCR5 cells using Lipofectamine LTX (Thermo Fisher Scientific, Schwerte, Germany) according to manufacturer’s instruction, cells stably express feline A3s were selected by 750 μg/ml G418 (Biochrom, GmbH) in the following 3 weeks. The A3s degradation experiments were performed in 24-well plates, 1 × 10^5^ 293T cells were transfected with 250 ng A3s expression plasmids together with 250 ng HIV-1, HIV-2, SIVmac and SIVsmm Vif expression plasmids or 20 ng codon-optimized FIV Vif expression plasmid, pcDNA3.1 (+) (Life Technologies) was used to fill the total plasmid to 500 ng. To produce FIV-luciferase viruses, 293T cells were co-transfected with 0.6 μg FIV packaging construct, 0.6 μg FIV-luciferase vector, 1 μg A3 expression plasmid, 0.1 μg VSV-G expression plasmid; in some experiments pcDNA3.1 (+) (Life Technologies) was used instead of Vif or A3 expression plasmids. For HIV-2 and SIVmac-luciferase transfections, 1.2 μg HIV-2-Luc and SIVmac-Luc plasmids were used instead of FIV plasmids. At 48 h post transfection, cells and supernatants were collected.

### Vif and A3 plasmids

FIV-34TF10 (codon-optimized), HIV-1, HIV-2, SIVmac and SIVsmm Vif genes were inserted into pcWPRE containing a C-terminal V5 tag [36]. HIV-1 Vif represents always HIV-1 Vif from clone NL4-3, except specifically stated LAI. HIV-1 Vif LAI is a gift from Viviana Simon and does not contain a protein tag [54]. pCPRΔ*env* FIV gag-pol plasmid that in addition expresses Vif was described previously [57]. FIV-Lion Vif gene (FIV_*Ple*_ subtype B, accession number EU117991) was synthesized and codon-optimized. FIV-Lion Vif expression plasmid was generated by cloning codon-optimized FIV-Lion Vif fragment containing a V5 tag into pcWPRE using EcoRI and NotI. All A3s are expressed a carboxy-terminal hemagglutinin (HA) tag. Domestic cat and big cat (*Pantherinae*) A3s were described previously [36]. Human A3C (HsaA3C) and feline A3Z2b (FcaA3Z2b) chimeras were made by overlapping extension PCR. HsaA3C/FcaA3Z2 chimera Z2C1, Z2C4 and Z2C5 contain residues 1–22, 1–131 and 1–154 of FcaA3Z2, respectively; the remaining C-terminal fragments are derived from human A3C. The 5′ and 3′ fragments were amplified separately by using primer pairs (Additional file 2: Table S1); two fragments were then mixed and amplified with the two external primers (Additional file 2: Table S1). To make HsaA3C/FcaA3Z2 chimera Z2C30, the first fragment was amplified by primers feApo3.fw and hufe3C 485.rv using chimera Z2C4 as a template, the second fragment was amplified by primers hufe3C 485.fw and HA-rv using FcaA3Z2 as a template, the two fragments were mixed and amplified with the two external primers. The FcaA3Z2b mutants were generated by fusion PCR using primer pairs described in Additional file 2: Table S1. The final products of HsaA3C/FcaA3Z2 chimeras and FcaA3Z2 mutants were cloned into pcDNA3.1 (+) using HindIII and XhoI restriction sites. The HsaA3H/FcaA3Z3 chimeras were constructed by the same method using primer pairs listed in Additional file 2: Table S2. To make FcaA3Z2bZ3-M, the PCR products of FcaA3Z2b DH-YN and FcaA3Z3 A65I + LI-AA were fused, and then inserted into pcDNA3.1 (+) by EcoRI and NotI restriction sites. The FcaA3Z2Z3 mutation constructs were generated by using the primers shown in Additional file 2: Table S3.

### Viruses and infection

To produce FIV single-cycle luciferase viruses (FIV-Luc), 293T cells were co-transfected with the replication deficient packaging construct pFP93, a gift from Eric M. Poeschla [85], which only expresses *gag*, *pol*, and *rev*; the FIV luciferase vector pLinSin [4]; a VSV-G expression plasmid pMD.G; FcaA3s expression plasmids; FIV Vif expression plasmid; or empty vector pcDNA3.1 (+). To produce SIV-Luc viruses, 293T cells were co-transfected with SIVmac-Luc (R-E-); or SIVmac-Luc (R-E-)Δ*vif* [63]; and FcaA3s expression plasmids. HIV-2-Luc was produced by co-transfecting 293T cells with HIV-2 packaging plasmid pHIV2Δ4 [86]; transfer vector plasmid HIV-2-luc (SV40) [64]; pMD.G, together with FcaA3s expression plasmids or empty vector pcDNA3.1 (+) and HIV-2 Vif-V5 expression plasmid or pcDNA3.1 (+) empty vector. The reverse transcriptase (RT) activity of FIV, SIVmac and HIV-2 were quantified by using the Cavidi HS lenti RT kit (Cavidi Tech, Uppsala, Sweden). For reporter virus infection, 293T cells were seeded in 96-well plate 1 day before transduction. After normalizing for RT activity, the same amounts of viruses were used for infection. Three days post transduction, firefly luciferase activity was measured with the Steadylite HTS reporter gene assay system (Perkin-Elmer, Cologne, Germany) according to the manufacturer’s instructions on a MicroLumat Plus luminometer (Berthold Detection Systems, Pforzheim, Germany). Each sample was performed transduction in triplicates; the error bar of each triplicate was shown. Replication-competent HIV-1 plasmids NL-BaL.*vif*_FIV_ were described previously [36]. NL-BaL.*vif*_FIV_ virus stocks were prepared by collecting the supernatant of transfected 293T cells. The kinetics of viral spreading replication was determined with HOS.CD4.CCR5.FcaA3s cells by infection with MOI 0.01 of NL-BaL.*vif*_FIV_. Spreading virus replication was quantified over 15 days by infecting 10 μl supernatant to TZM-bl cells. All experiments were repeated independently at least three times.

### Immunoblot analysis

Transfected 293T cells were lysed in radioimmunoprecipitation assay (RIPA) buffer (25 mM Tris–HCl [pH7.6], 150 mM NaCl, 1 % NP-40, 1 % sodium deoxycholate, 0.1 % sodium dodecyl sulfate [SDS], protease inhibitor cocktail set III [Calbiochem, Darmstadt, Germany]). The expression of FcaA3s and lentivirus Vif were detected by mouse anti-hemagglutinin (anti-HA) antibody (1:7500 dilution, MMS-101P; Covance, Münster, Germany) and mouse anti-V5 antibody (1:4500 dilution, MCA1360, ABDserotec, Düsseldorf, Germany) separately, the tubulin and SIV capsid protein were detected using mouse anti-α-tubulin antibody (1:4000, dilution, clone B5-1-2; Sigma-Aldrich, Taufkirchen, Germany), HIV Vif LAI was detected by HIV-1 Vif monoclonal antibody (#319) (NIH AIDS Reagent Program [87]) and mouse anti-capsid p24/p27 MAb AG3.0 (1:50 dilution [88]) separately, followed by horseradish peroxidase-conjugated rabbit anti-mouse antibody (α-mouse-IgG-HRP; GE Healthcare, Munich, Germany), and developed with ECL chemiluminescence reagents (GE Healthcare). Encapsidation of FcaA3 proteins into FIV particles: HEK293T cells were transfected with 600 ng pFP93, 600 ng of pLinSin, 100 ng pMD.G and 1000 ng of FcaA3 constructs. Viral supernatants were collected 48 h later, overlaid on 20 % sucrose and centrifuged for 4 h at 14,800 rpm in a table top centrifuge. Viral pellet was resuspended in RIPA buffer, boiled at 95 °C for 5 min with Roti load reducing loading buffer (Carl Roth, Karlsruhe, Germany) and resolved on a SDS-PAGE gel. The FcaA3s and tubulin proteins were detected as the above method. VSV-G and FIV p24 proteins were detected using mouse anti-VSV-G antibody (1:10,000 dilution; clone P5D4; Sigma-Aldrich) and mouse anti-FIV p24 antibody (1:2000 dilution; clone PAK3-2C1; NIH AIDS REPOSITORY) separately, followed by horseradish peroxidase-conjugated rabbit anti-mouse antibody (α-mouse-IgG-HRP; GE Healthcare, Munich, Germany), and developed with ECL chemiluminescence reagents (GE Healthcare).

### Immunofluorescence and flow cytometry

HOS cells grown on polystyrene coverslips (Thermo Fisher Scientific, Langenselbold, Germany) were transfected with expression plasmids for FcaA3 wild-type and mutants or together with FIV Vif-TLQAAA using Lipofectamine LTX (Life Technologies). At day one post transfection, cells were fixed in 4 % paraformaldehyde in PBS for 30 min, permeabilized in 0.1 % Triton X-100 in PBS for 15 min, incubated in blocking buffer (FBS in PBS) for 1 h, and then cells were stained by mouse anti-HA antibody in a 1:1000 dilution in blocking solution for 1 h. Donkey anti-mouse Alexa Fluor 488 (Life Technologies) was used as a secondary antibody in a 1:300 dilution in blocking solution for 1 h. FIV Vif-TLQAAA was stained by rabbit anti-V5 antibody in a 1:1000 dilution in blocking solution for 1 h. Donkey anti-rabbit Alexa Fluor 594 (Life Technologies) was used as a secondary antibody in a 1:300 dilution in blocking solution for 1 h. Finally, DAPI was used to stain nuclei for 2 min. The images were captured by using a 40× objective on a Zeiss LSM 510 Meta laser scanning confocal microscope (Carl Zeiss, Cologne, Germany). To analyze CD4 and CCR5 expression level of HOS.CD4.CCR5.FcaA3s, cells were stained by α-hCD4 PE mouse IgG1_k_ (Dako, Hamburg, Germany) and α-hCCR5 FITC (BD Bioscience, Heidelberg, Germany) separately according to the manufacturer’s instruction. The measurement was carried out by BD FACSanto (BD Bioscience). Data analysis was done with the Software FlowJo version 7.6 (FlowJo, Ashland, USA).

### Immunoprecipitation

To determine Vif and A3 binding, 293T cells were co-transfected with 1 μg FIV Vif TLQAAA-V5 and 1 μg FcaA3 wild-type or mutants or pcDNA3.1 (+). 48 h later, the cells were lysed in IP-lysis buffer (50 mM Tris/HCl pH 8, 1 mM PMSF, 10 % Glycerol, 0.8 % NP-40, 150 mM NaCl, and protease inhibitor cocktail set III (Calbiochem, Darmstadt, Germany). The lysates were cleared by centrifugation. The supernatant were incubated with 20 μl α-HA Affinity Matrix Beads (Roche) at 4 °C for 2 h. The samples were washed 5 times with lysate buffer on ice. Bound proteins were eluted by boiling the beads for 5 min at 95 °C in SDS loading buffer. Immunoblot analysis and detection were done as described.

### 3D-PCR

293T cells (5 × 10^5^ cells/well in a 6-well plate) were transfected with 600 ng pFP93, 600 ng pLinSin, 100 ng pMD.G and 1000 ng FcaA3s expression plasmids or pcDNA3.1 (+) as a control. 48 h later, the viral supernatant was harvested, filtered (0.45 µm) and treated with DNase I (Life Technologies) at 37 °C for 1 h. 200 μl of supernatant was used for infecting 293T cells. 12 h post transduction, 293T cells were washed with PBS and DNA was isolated using DNeasy blood and tissue kit (Qiagen, Hilden, Germany). A 714-bp fragment of within the spliced luciferase gene was amplified using the primers 5′-GATATGTGGATTTCGAGTCGTC-3′ and 5′-GTCATCGTCTTTCCGTGCTC-3′. For selective amplification of the hypermutated products, the PCR denaturation temperature were lowered stepwise from 87.6 to 83.5 °C (83.5, 84.2, 85.2, 86.3, 87.6 °C) using a gradient thermocycler. The PCR parameters were as follows: (1) 95 °C for 5 min; (2) 40 cycles, with 1 cycle consisting of 83.5–87.6 °C for 30 s, 55 °C for 30 s, 72 °C for 1 min; (3) 10 min at 72 °C. PCRs were performed with recombination *Taq* DNA polymerase (Thermo Fisher Scientific).

### Purification of GST tagged proteins and pull down assay

Feline A3Z2 and A3Z3 coding sequences were cloned in pGEX-6P2 vector (GE healthcare) with a C terminal HA tag to produce fusion proteins GST-FcaA3Z2-HA and GST-FcaA3Z3-HA (PCR primer in Additional file 2: Table S4). GST alone and fusion proteins were overexpressed in *E. coli* Rosetta (DE3) cells (EMD Millipore, Darmstadt, Germany) and purified by affinity chromatography using Glutathione Sepharose 4B beads (GE healthcare). After the culture of transformants until 0.6 OD_600_, cells were induced with 1 mM isopropyl-beta-d-thiogalactopyranoside (IPTG) and 1 µM ZnSO_4_ and cultured at 18 °C overnight. GST and Feline A3Z2/Z3 harboring cells were washed with PBS and lysed with 1× Bug buster protein extraction reagent (EMD Millipore) containing 50 mM Tris (pH 7.0), 10 % glycerol, and 1 M NaCl clarified by centrifugation and the soluble protein fraction was mixed with pre-equilibrated glutathione Sepharose beads. After 3 h incubation at 4 °C in end-over-end rotation, the beads were washed thrice with wash buffer containing 50 mM Tris (pH 8.0), 10 % glycerol and 500 mM NaCl and a single wash with the mild lysis buffer (50 mM Tris (pH 8), 1 mM PMSF, 10 % glycerol, 0.8 % NP-40, 150 mM NaCl and 1× complete protease inhibitor). These GST protein bound beads are used for the subsequent binding assay. GST pull down assay to detect direct binding with Vif of FIV: The protocol of protein–protein interactions was adapted from a previously described procedure [89]. HEK293T cells were transfected with 1.5 µg of FIV Vif-V5 coding plasmid and incubated for 48 h. Soluble protein fraction of HEK293T cells were obtained by lysing the cells with mild lysis buffer (50 mM Tris (pH 8), 1 mM PMSF, 10 % glycerol, 0.8 % NP-40, 150 mM NaCl, and 1× complete protease inhibitor (Calbiochem) and a 30 min centrifugation at 14,800 rpm. A fraction of the supernatant was kept for immunoblots; remaining lysates were equally added on the bead samples GST, GST-FcaA3Z2-HA and GST-FcaA3Z3-HA and incubated overnight at 4 °C in end-over-end rotation. Next day, the beads were washed thrice with the mild lysis buffer and the GST protein and protein complexes were eluted by adding wash buffer containing 25 mM reduced glutathione. A fraction of the eluted proteins (equal amount) were boiled at 95 °C for 5 min with Roti load reducing loading buffer (Carl Roth) and resolved on a SDS-PAGE gel. FIV Vif and GST-FcaA3s were detected by anti-V5 and -HA antibody, respectively. Coomassie brilliant blue stained gel was also added to show the purity of GST and FcaA3 fusion proteins.

### Evolutionary analyses

The initial set of A3 sequences was taken from Münk and coworkers [3]. These sequences were used as seeds for BLASTn, tBLASTn and BLAT searches to recover additional A3 sequences from genomes in Carnivora. The final dataset (closed on November 2015) contained four A3Z1 sequences from four Caniformia species, six A3Z2 sequences from six Caniformia species, eleven A3Z2 sequences from six Feliformia species, ten A3Z3 sequences from five Caniformia species and five A3Z3 sequences from five Feliformia species. Sequences were aligned at the amino acid level with MUSCLE [90]. The final alignment encompassed 629 and 269 alignment patterns at the nucleotide level and at the amino acid level, respectively. Phylogenetic inference was performed with RAxML_v8.2 [91, 92] using the GTR + 4Γ model at the nucleotide level and LG + Γ model at the amino acid level, the model choice done after initial maximum likelihood searches with RAxML. Additional phylogenetic inference was performed separately for the A3Z2 and A3Z3 genes using the same settings. In all cases, no significant differences between amino acid and nucleotide tree topologies were observed using the Shimodaira–Hasegawa test [93]. Phylogenetic supernetworks were constructed with SplitsTree_v4 [94] using 1000 either nucleotide or amino acid bootstrapped maximum likelihood trees. Selection on individual codons was inferred under a Bayesian framework with SELECTON V2.4 (http://selecton.tau.ac.il/) [95] contrasting the M8 and M8a models, and with DATAMONKEY (http://www.datamonkey.org/) using the Random Effects Likelihood (REL) model [96].

### Statistical analysis

Data are represented as the mean with SD in all bar diagrams. Statistically significant differences between two groups were analyzed using the unpaired Student’s t test with GraphPad Prism version 5 (GraphPad software, San Diego, CA, USA). Validity of the null hypothesis was verified with significance level at α value = 0.05.

### Homology modelling of feline A3Z2Z3 protein

The homology modeling of the linker region of the feline A3Z2Z3 was performed in several steps: First, the in-house meta-tool TopModel [59, 60] was used to compute a consensus alignment for the feline A3 sequences to the structural model of the human A3G [58] using 13 different alignment programs (Additional file 2: Table S5). From the consensus alignment, the feline A3 linker was identified and then submitted to TopModel for automated structure prediction using eight state-of-the-art threading programs (Additional file 2: Table S5). The identified templates (2YS9, chain A (19.4 %); 2MMB, chain A (17.1 %); 2DA4, chain A (14.7 %); 2LFB, chain A (9.2 %) and 1FTZ, chain A (12.9 %); sequence identities with respect to the linker are given in parentheses) were aligned to the linker sequence with TopModel using threading, sequence, and structural alignment programs, to produce a large alignment ensemble from every combination of the top three ranked templates and the target sequence. These alignments were modeled using Modeller9.1 [97], refined with RASP [98], and ranked using the in-house meta-tool for model quality assessment TopScore (D. Mulnaes, H. Gohlke, unpublished results), which combines quality assessments from eight different model quality assessment programs (Additional file 2: Table S5). The top ranked models for each template combination were refined with ModRefiner [99] and used as templates for a second round of modeling where bad scoring regions were removed. The resulting models were re-ranked and refined, and the top ranking model was selected as the linker representative. The model of the rest of the feline A3Z2Z3 was made with TopModel in a similar fashion using the feline–human consensus alignment. The linker domain was manually positioned near the linker region gap, unstructured parts were connected to the rest of the feline A3Z2Z3 and minimized using the MAB force field [100] as implemented in Moloc, thereby keeping all other protein atoms fixed.

### Homology modelling of human A3H and feline A3Z2b and A3Z3 proteins

The models of the three proteins were built using the default settings in TopModel and all possible combinations of the top three ranked templates in each case. For the human A3H model, the templates were: PDB ID 4 J4 J, chain A, 35 % identity/96 % coverage; 2KBO, chain A, 37/95 %; 2RPZ, chain A, 30/94 %, resulting in a model with 84 % accuracy according to TopScore. For the feline A3Z2b model, the templates were: 3VM8, chain A, 42/94 %; 2KBO, chain A, 39/94 %; 1M65, chain A, 10/87 %, resulting in a model with 88 % accuracy according to TopScore. For the feline A3Z3 model, the templates were: 4J4J, chain A, 31/91 %; 2KBO, chain A, 36/90 %; 2RPZ, chain A, 24/92 %, resulting in a model with 84 % accuracy according to TopScore.

## Additional files

10.1186/s12977-016-0274-9. *Cellular localization of feline A3*s *and FIV Vif.* HOS cells were transfected with FcaA3Z2b, FcaA3Z3, or FcaA3Z2Z3 (all with HA-tag), together with FIV Vif-TLQAAA. To detect A3 (green) immunofluorescence, staining was performed with an anti-HA antibody. To detect FIV Vif (red) immunofluorescence, staining was performed with an anti-V5 antibody. Nuclei (blue) were visualized by DAPI staining. **Figure S2**. *Comparison of protein sequences of A3*s *and Vif.* (A, B) The sequence alignment of (A) FcaA3Z2 (FcaA3Z2b), (B) FcaA3Z3 and big cat A3 proteins. The D165-H166 and L40-I41 + A65 domains that are essential for FIV Vif induced degradation are marked by red boxes. (C) Sequence alignment of domestic cat FIV Vif (FIVfca subtype 34TF10) and lion FIV (FIVple subtype E) Vif. The C187 and C190 that are essential for induced FcaA3s degradation and marked the presumed BC box (TLQ/SLQ) marked by red boxes. (D) Sequence alignment of HIV-1 (strain NL4-3) and HIV-2 (strain RodA) Vif. The CUL5 box (HCCH) and BC box (SLQ) were marked by red boxes. Pti, Ple, Lly and Pco represent *Panthera tigris corbetti*; *Panthera leo bleyenberghi*; *Lynx lynx*; *Puma concolor*. **Figure S3**. *Evolutionary supernetwork of A3 sequences retrieved from carnivores.* The network was constructed with SplitsTree_v4 using 1,000 maximum likelihood bootstrapped trees created with RAxML_v8.2. Scale bar is given in substitutions per site. The approximate position of the root obtained using maximum likelihood inference with all A3Z1, A3Z2 and A3Z3 sequences from carnivores is indicated in grey. (A) The evolutionary distances among A3Z3 sequences within the two in-paralogs within Caniformia (upper branches and left branch) and within Feliformia (right branches) are indicated as overall average pairwise nucleotide distance ± bootstrap standard error estimate. For each tip, the actual sequence orthologous to positions 38-44 in the *F. catus* A3Z3 gene are given in parentheses. The inset displays the evolutionary relationships among the Carnivora species for which we have identified A3Z3 paralogs. (B) The evolutionary distances among A3Z2 sequences within Caniformia (upper branches) and within Feliformia (lower branches) are indicated as overall average pairwise nucleotide distance ± bootstrap standard error estimate. For each tip, the actual sequence orthologous to positions 165-170 in the *F. catus* A3Z2 genes are given in parentheses. **Figure S4**. *The mutations in FcaA3 that cause resistance to FIV Vif do not alter the cellular distribution of FcaA3*s. HOS cells were transfected with FcaA3Z2 (FcaA3Z2b), FcaA3Z2.DH-YN, FcaA3Z3, FcaA3Z3.A65I + LI-AA, FcaA3Z2Z3 or FcaA3Z2Z3-M (all with HA-tag). To detect A3 (green) immunofluorescence, staining was performed with an anti-HA antibody. Nuclei (blue) were visualized by DAPI staining. **Figure S5**. *Sequence alignment of feline APOBEC3.* Sequence alignment of feline A3Z2Z3 and human A3G as generated by the TopModel approach. The Z2 and Z3 domains are underlined in yellow and blue, respectively. The sequence of the linker domain is underlined in magenta. Helical regions and β-strands are depicted as red helices and green arrows, respectively. In addition, the alignments of three template structures used to model the structure of the linker domain are given (PDB IDs 2YS9, 2DA4, 2MMB). **Figure S6**. *Expression of CD4 and CCR5 receptors on the surface of HOS (red) and HOS.CD4.CCR5 cells (blue) expressing feline A3Z2Z3 or A3Z2Z3*-*M.* CD4 and CCR5 were detected by flow cytometry and anti-CD4 and anti-CCR5 antibodies. Numbers indicate the percentage of positive cells. HOS cells served as background control. **Figure S7**. *The mutated FcaA3*s *are encapsidated and inhibit FIV by cytidine deamination.* (A) The mutated FcaA3s can inhibit the infectivity of FIVΔ*vif* reporter viruses. 293T cells were co-transfected with plasmids for FIVΔ*vif* luciferase together with FcaA3s. 48 h later, supernatant normalized for reverse transcriptase activity was used to transduce 293T cells. Luciferase activity was determined two days post transduction. Asterisks represent statistically significant differences: ***, *p* < 0.001; **, 0.001 < *p* < 0.01; *, 0.01 < *p* < 0.05; ns, *p* > 0.05 [Dunnett *t* test]. (B) Immunoblot of FIV producer cells and VLPs used for (A). Encapsidation of wild-type and mutated feline A3s into FIVΔ*vif* virus like particles (VLPs), A3 proteins were detected by anti-HA antibody. Tubulin detection for equal loading of cell lysate was done using anti-tubulin, for demonstration of equal loading of FIV VLPs VSV-G and FIV p24 proteins were detected by anti-VSV-G and anti-FIV p24 antibodies separately. (C) Encapsidated wild-type and mutated FcaA3s deaminate cytidines FIV genomes. FIVΔ*vif* was produced in the absence and presence of wild-type and mutant FcaA3s (FcaA3Z3, FcaA3Z3.A65I + LI-AA, FcaA3Z2Z3, FcaA3Z2Z3-M) or HsaA3G. The vector particles were used to infect 293T cells. 12 h later, the total cellular DNA was extracted and differential DNA denaturation PCR (3D-PCR) was performed. Td: denaturing temperature. **Figure S8**. *Vif titration on FcaA3 linker mutants.* Co-transfection of increasing amounts of expression plasmids for (A) HIV-2 Vif and (B) SIVmac Vif with constant amounts of the indicated A3 expression plasmids. The expression of FcaA3s and Vifs were analyzed by anti-HA and anti-V5 antibodies, respectively. Cell lysates were also analyzed for equal amounts of total proteins using anti-tubulin antibody. **Figure S9**. *Expression and encapsidation of feline A3 linker mutants using (A) FIVΔvif and (B) SIVmacΔvif.* Immunoblots of corresponding experiments shown in Fig. 10C and 10D. Immunoblots of lysates of virus producer cells (cell) and virus particles (VLP). A3s were detected by anti-HA antibodies, cell lysates were also analyzed for equal amounts of total proteins using anti-tubulin antibody and VLP lysates using anti-VSV-G antibody. **Figure S10.** *HIV*-*1 Vif cannot target the “YYFWDPN/DY” domain in FcaA3.* (A) CO-IP of feline A3Z2Z3 (HA tag) with either HIV-1 Vif (V5 tag) or FIV Vif (TLQAAA mutant, V5 tag). A3Z2Z3 immune precipitated and detected by anti-HA antibody, co-precipitated Vif was detected by anti-V5 antibody. (B) Comparison of the “YYFWDPN/DY” domain in HsaA3G and FcaA3Z2Z3 and derived mutations generated in FcaA3Z2Z3, the mutated residues shown in bold. (C) FcaA3Z2Z3 mutants were investigated for being sensitive for degradation by HIV-1 Vif. Expression plasmids of FcaA3Z2Z3 mutants of HsaA3G were co-transfected together with HIV-1 Vif into 293T cells. 48 h later, Cell lysates were used to detect the expression of FcaA3Z2Z3 and HIV-1 Vif by anti-HA and anti-V5 antibodies, respectively. Cell lysates were also analyzed for equal amounts of total proteins using anti-tubulin antibody.
10.1186/s12977-016-0274-9. Primer list used for HsaA3C/FcaA3Z2 chimeras and FcaA3Z2 mutants. **Table S2**. Primer list used for HsaA3H/FcaA3Z3 chimeras and FcaA3Z3 mutants. **Table S3**. Primer list used for FcaA3Z2Z3 mutants. **Table S4**. Primer used to clone GST fusion constructs. **Table S5**. The software used in TopModel for threading, alignment and model quality estimation^a^.

## Abbreviations

FIV: feline immunodeficiency virus
HIV: human immunodeficiency virus
SIV: simian immunodeficiency virus
APOBEC3: apolipoprotein B mRNA editing enzyme, catalytic polypeptide-like
Vif: viral infectivity factor
UTR: untranslated region
