# Supplementary material for: Determinants of FIV and HIV Vif sensitivity of feline APOBEC3 restriction factors
Source: Retrovirology. 2016 Jul 1;13:46. doi: 10.1186/s12977-016-0274-9 (PMC4930625; doi:10.1186/s12977-016-0274-9)
Supplement: Supplementary file 1 — 10.1186/s12977-016-0274-9. Cellular localization of feline A3s and FIV Vif. HOS cells were transfected with FcaA3Z2b, FcaA3Z3, or FcaA3Z2Z3 (all with HA-tag), together with FIV Vif-TLQAAA. To detect A3 (green) immunofluorescence, staining was performed with an anti-HA antibody. To detect FIV Vif (red) immunofluorescence, staining was performed with an anti-V5 antibody. Nuclei (blue) were visualized by DAPI staining. Figure S2. Comparison of protein sequences of A3s and Vif. (A, B) The sequence alignment of (A) FcaA3Z2 (FcaA3Z2b), (B) FcaA3Z3 and big cat A3 proteins. The D165-H166 and L40-I41 + A65 domains that are essential for FIV Vif induced degradation are marked by red boxes. (C) Sequence alignment of domestic cat FIV Vif (FIVfca subtype 34TF10) and lion FIV (FIVple subtype E) Vif. The C187 and C190 that are essential for induced FcaA3s degradation and marked the presumed BC box (TLQ/SLQ) marked by red boxes. (D) Sequence alignment of HIV-1 (strain NL4-3) and HIV-2 (strain RodA) Vif. The CUL5 box (HCCH) and BC box (SLQ) were marked by red boxes. Pti, Ple, Lly and Pco represent Panthera tigris corbetti; Panthera leo bleyenberghi; Lynx lynx; Puma concolor. Figure S3. Evolutionary supernetwork of A3 sequences retrieved from carnivores. The network was constructed with SplitsTree_v4 using 1,000 maximum likelihood bootstrapped trees created with RAxML_v8.2. Scale bar is given in substitutions per site. The approximate position of the root obtained using maximum likelihood inference with all A3Z1, A3Z2 and A3Z3 sequences from carnivores is indicated in grey. (A) The evolutionary distances among A3Z3 sequences within the two in-paralogs within Caniformia (upper branches and left branch) and within Feliformia (right branches) are indicated as overall average pairwise nucleotide distance ± bootstrap standard error estimate. For each tip, the actual sequence orthologous to positions 38-44 in the F. catus A3Z3 gene are given in parentheses. The inset displays the evolution [file 12977_2016_274_MOESM1_ESM.pptx]

## Slide 1
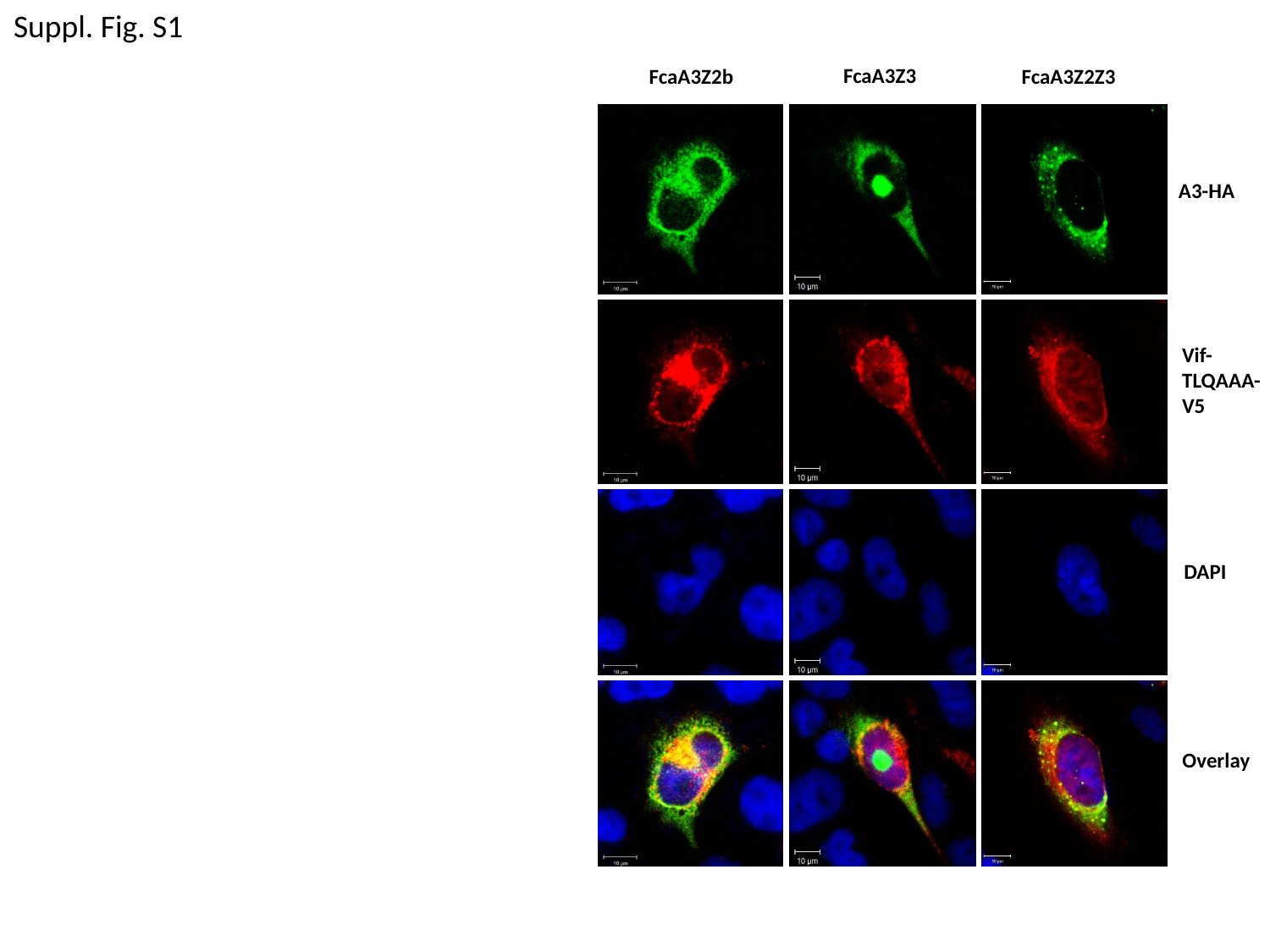

Suppl. Fig. S1
FcaA3Z3
FcaA3Z2b
FcaA3Z2Z3
A3-HA
Vif-TLQAAA-V5
DAPI
Overlay

## Slide 2
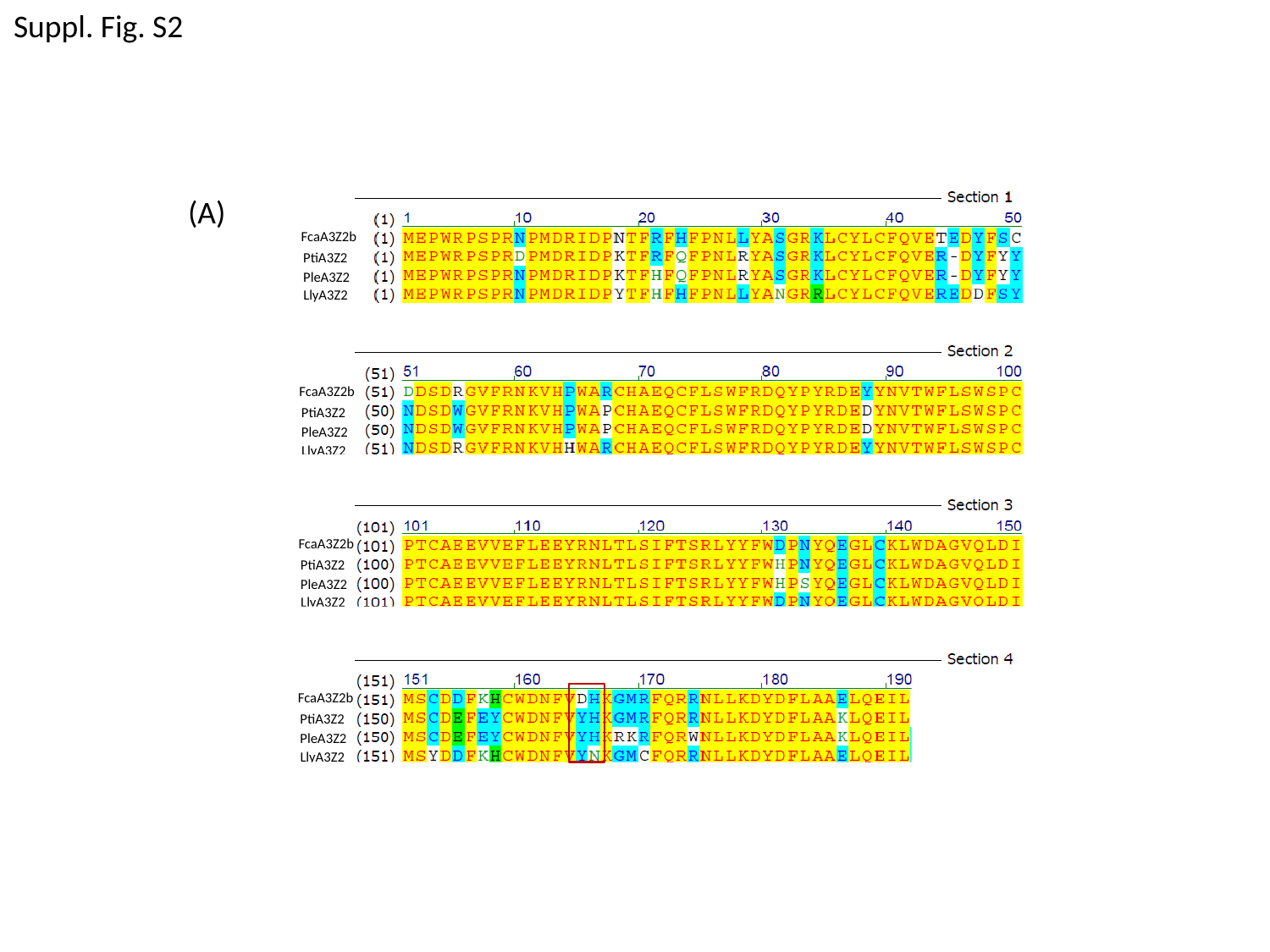

Suppl. Fig. S2
(A)
FcaA3Z2b
PtiA3Z2
PleA3Z2
LlyA3Z2
PcoA3Z2
FcaA3Z2b
PtiA3Z2
PleA3Z2
LlyA3Z2
PcoA3Z2
FcaA3Z2b
PtiA3Z2
PleA3Z2
LlyA3Z2
PcoA3Z2
FcaA3Z2b
PtiA3Z2
PleA3Z2
LlyA3Z2
PcoA3Z2

## Slide 3
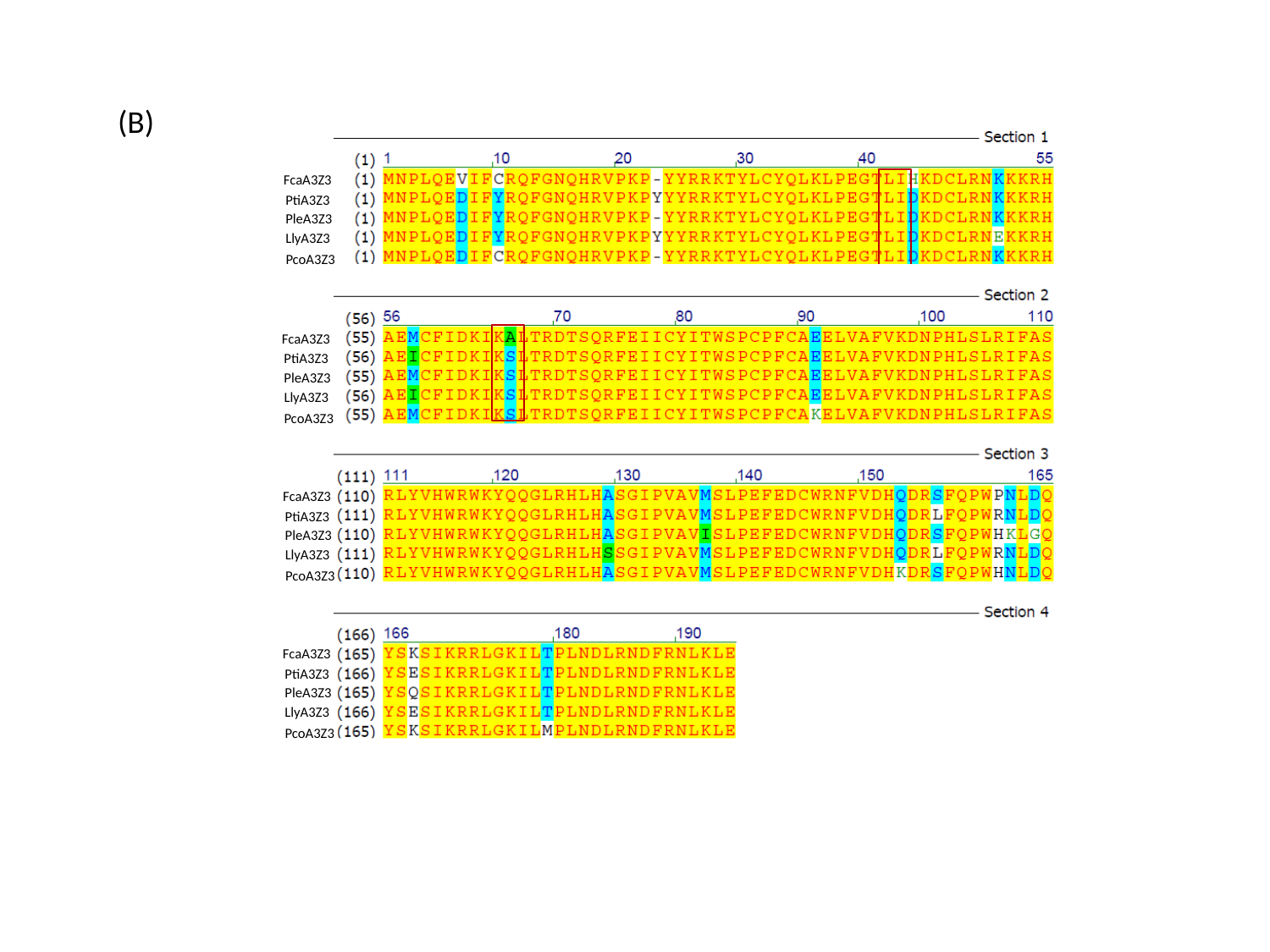

(B)
FcaA3Z3
PtiA3Z3
PleA3Z3
LlyA3Z3
PcoA3Z3
FcaA3Z3
PtiA3Z3
PleA3Z3
LlyA3Z3
PcoA3Z3
FcaA3Z3
PtiA3Z3
PleA3Z3
LlyA3Z3
PcoA3Z3
FcaA3Z3
PtiA3Z3
PleA3Z3
LlyA3Z3
PcoA3Z3

## Slide 4
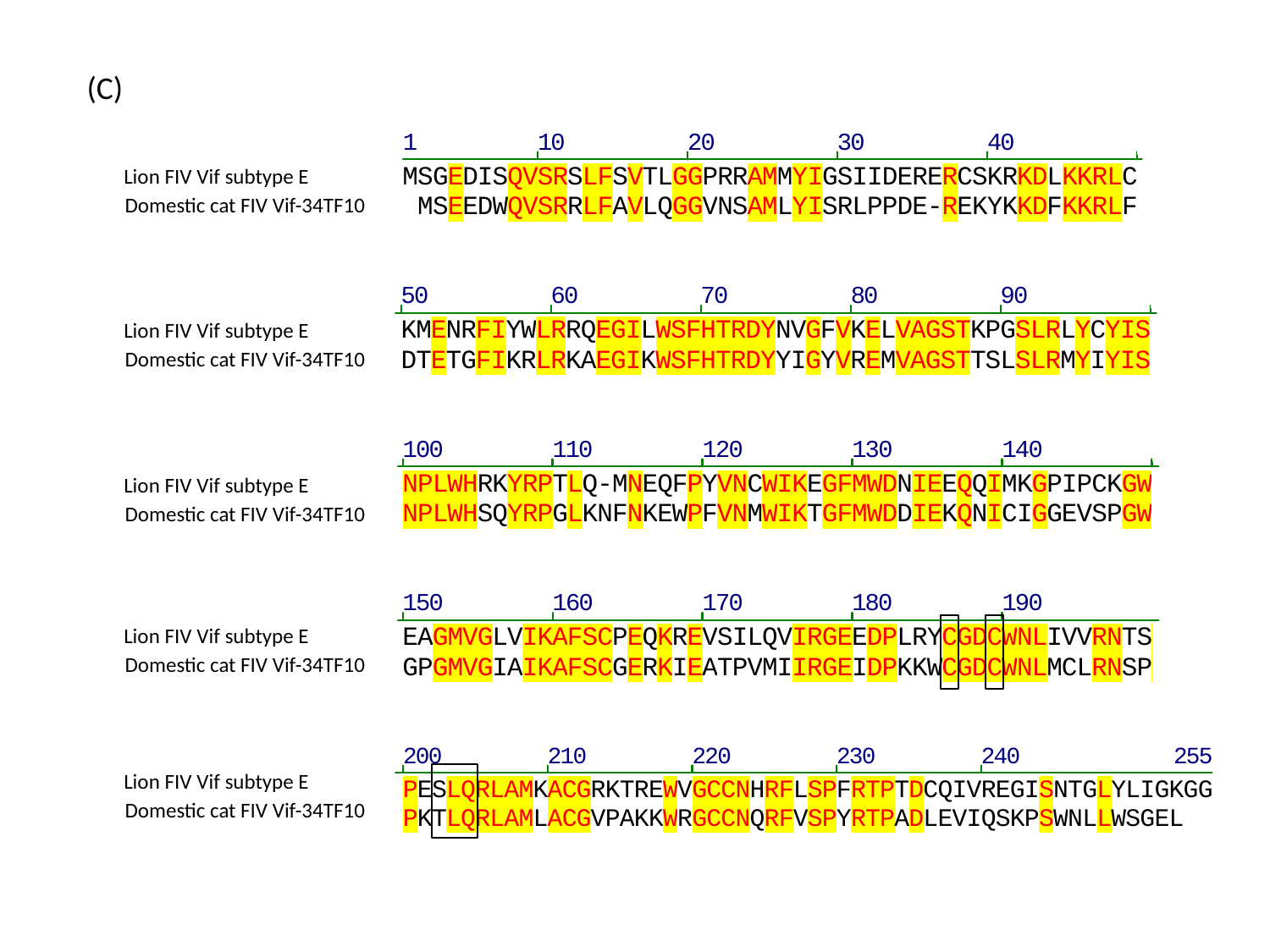

(C)
Lion FIV Vif subtype E
Domestic cat FIV Vif-34TF10
Lion FIV Vif subtype E
Domestic cat FIV Vif-34TF10
Lion FIV Vif subtype E
Domestic cat FIV Vif-34TF10
Lion FIV Vif subtype E
Domestic cat FIV Vif-34TF10
Lion FIV Vif subtype E
Domestic cat FIV Vif-34TF10

## Slide 5
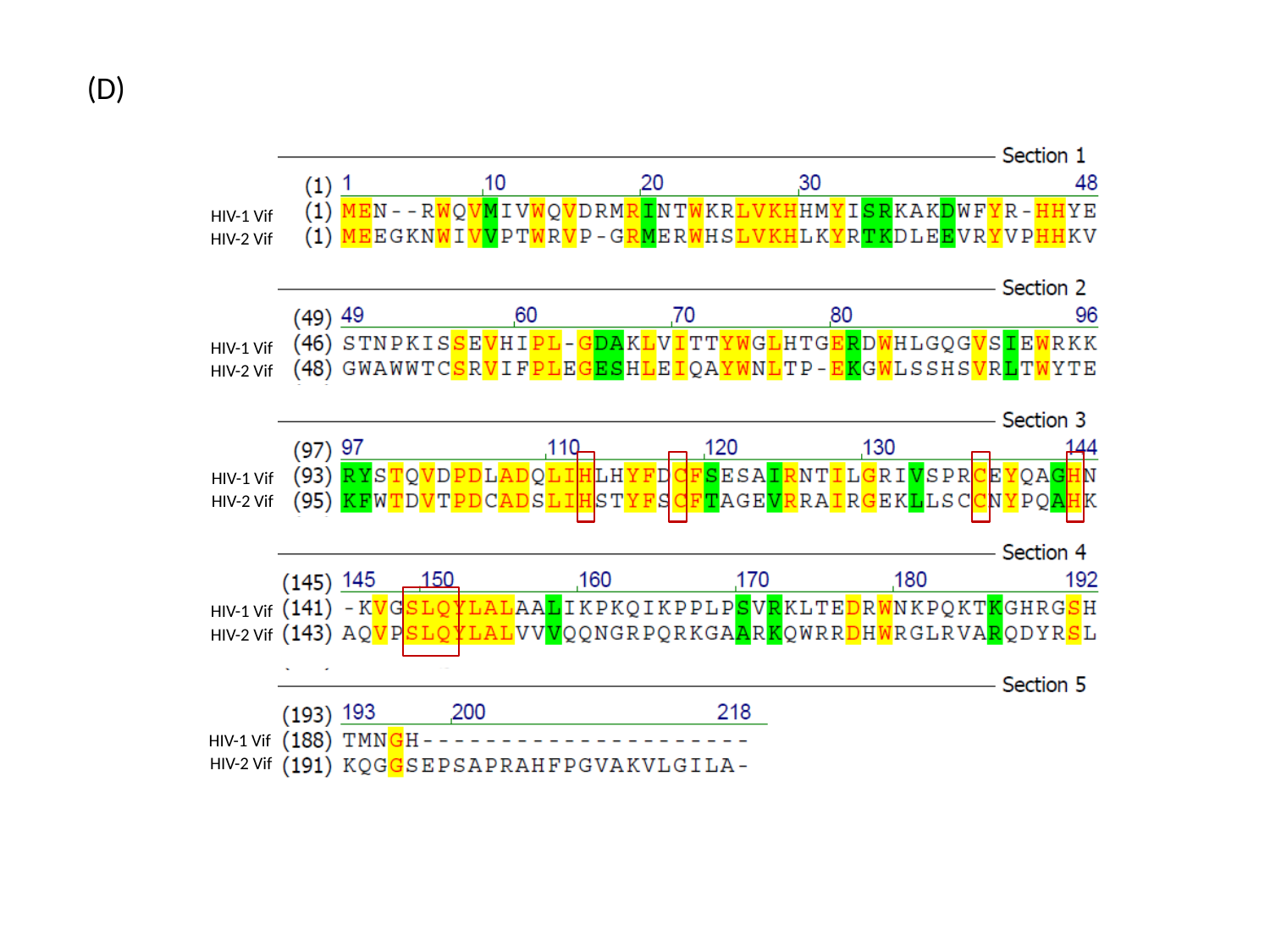

(D)
HIV-1 Vif
HIV-2 Vif
HIV-1 Vif
HIV-2 Vif
HIV-1 Vif
HIV-2 Vif
HIV-1 Vif
HIV-2 Vif
HIV-1 Vif
HIV-2 Vif

## Slide 6
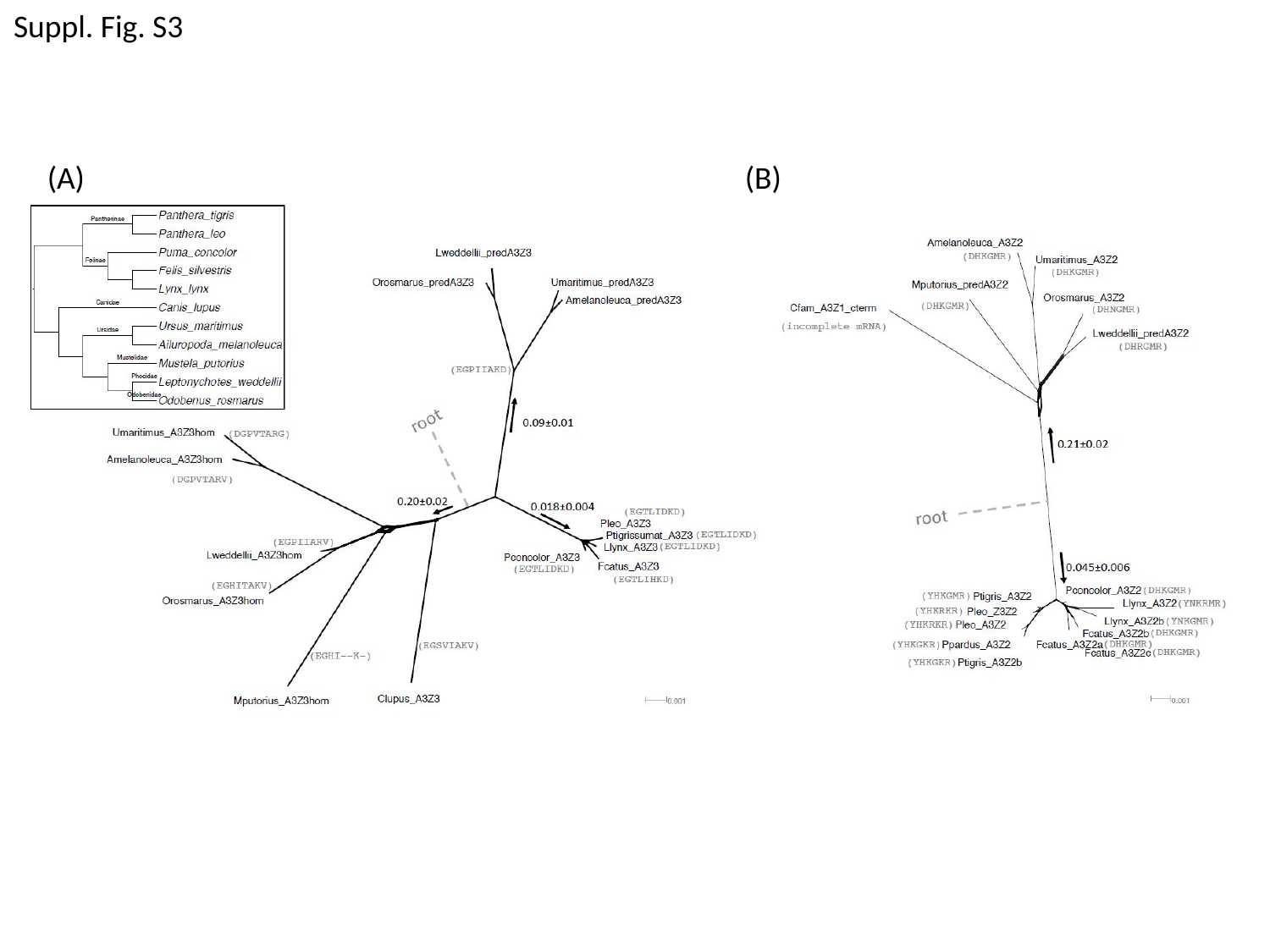

Suppl. Fig. S3
(A)
(B)

## Slide 7
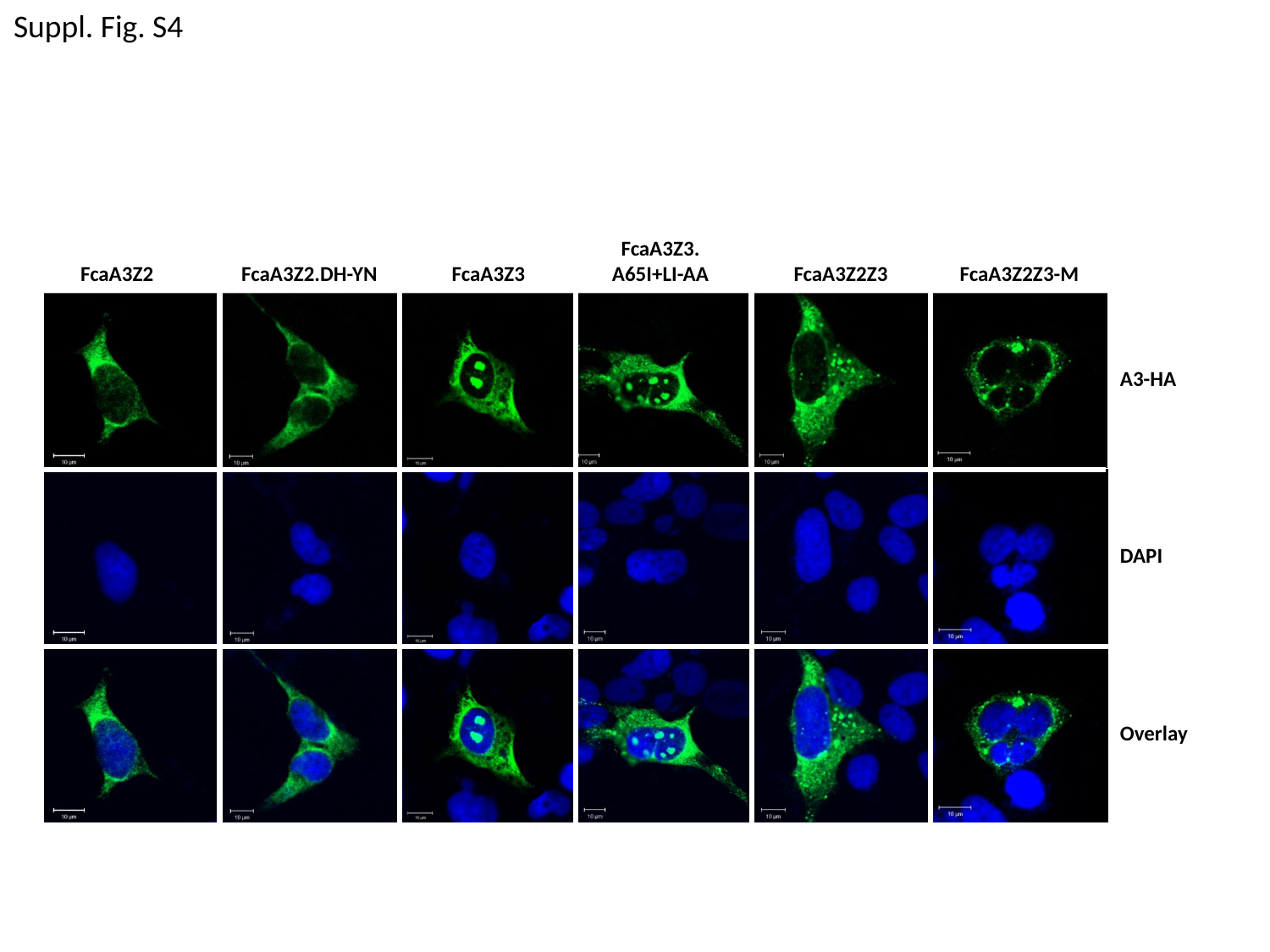

Suppl. Fig. S4
FcaA3Z3.
A65I+LI-AA
FcaA3Z2
FcaA3Z2.DH-YN
FcaA3Z3
FcaA3Z2Z3
FcaA3Z2Z3-M
A3-HA
DAPI
Overlay

## Slide 8
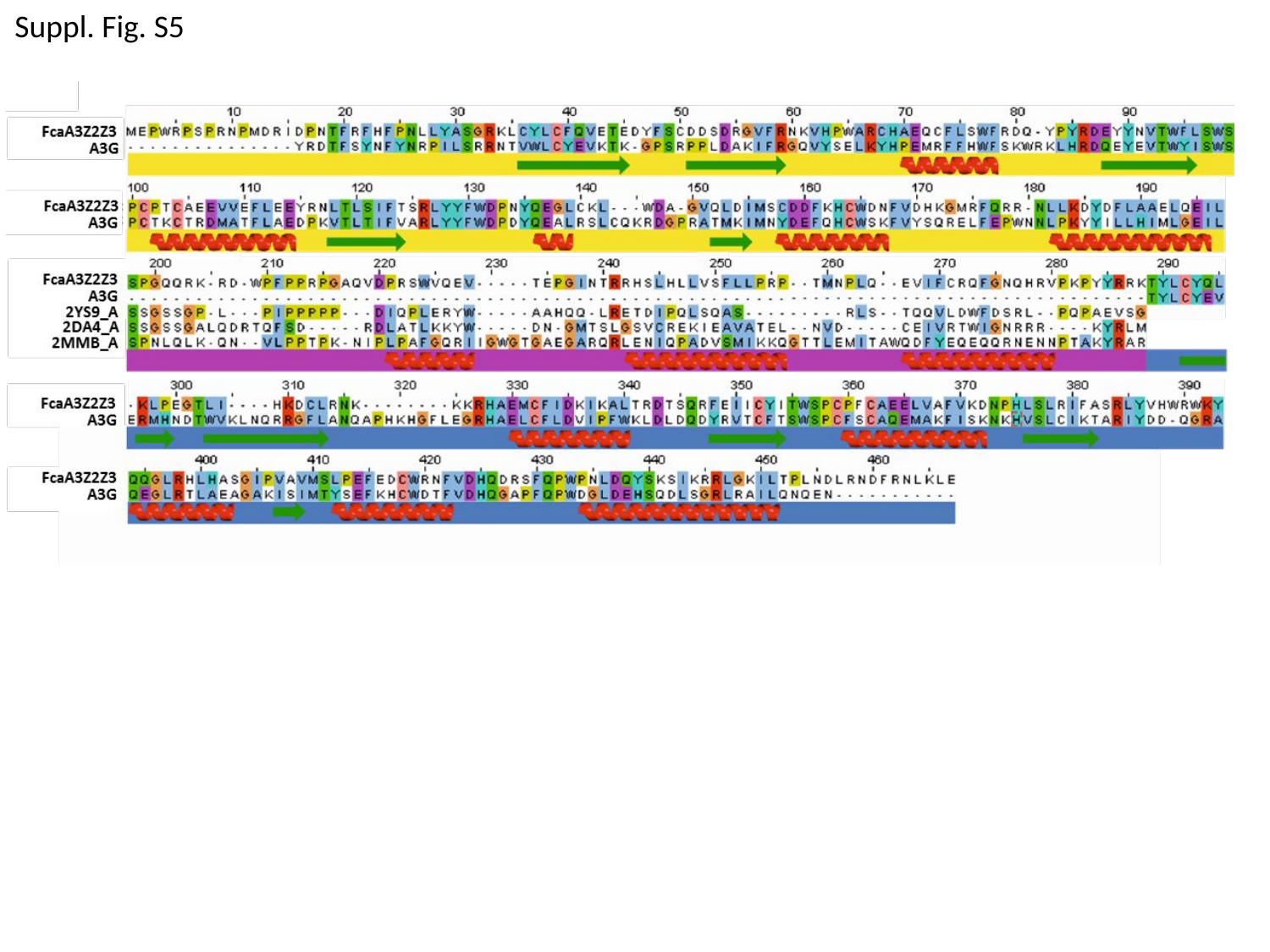

Suppl. Fig. S5

## Slide 9
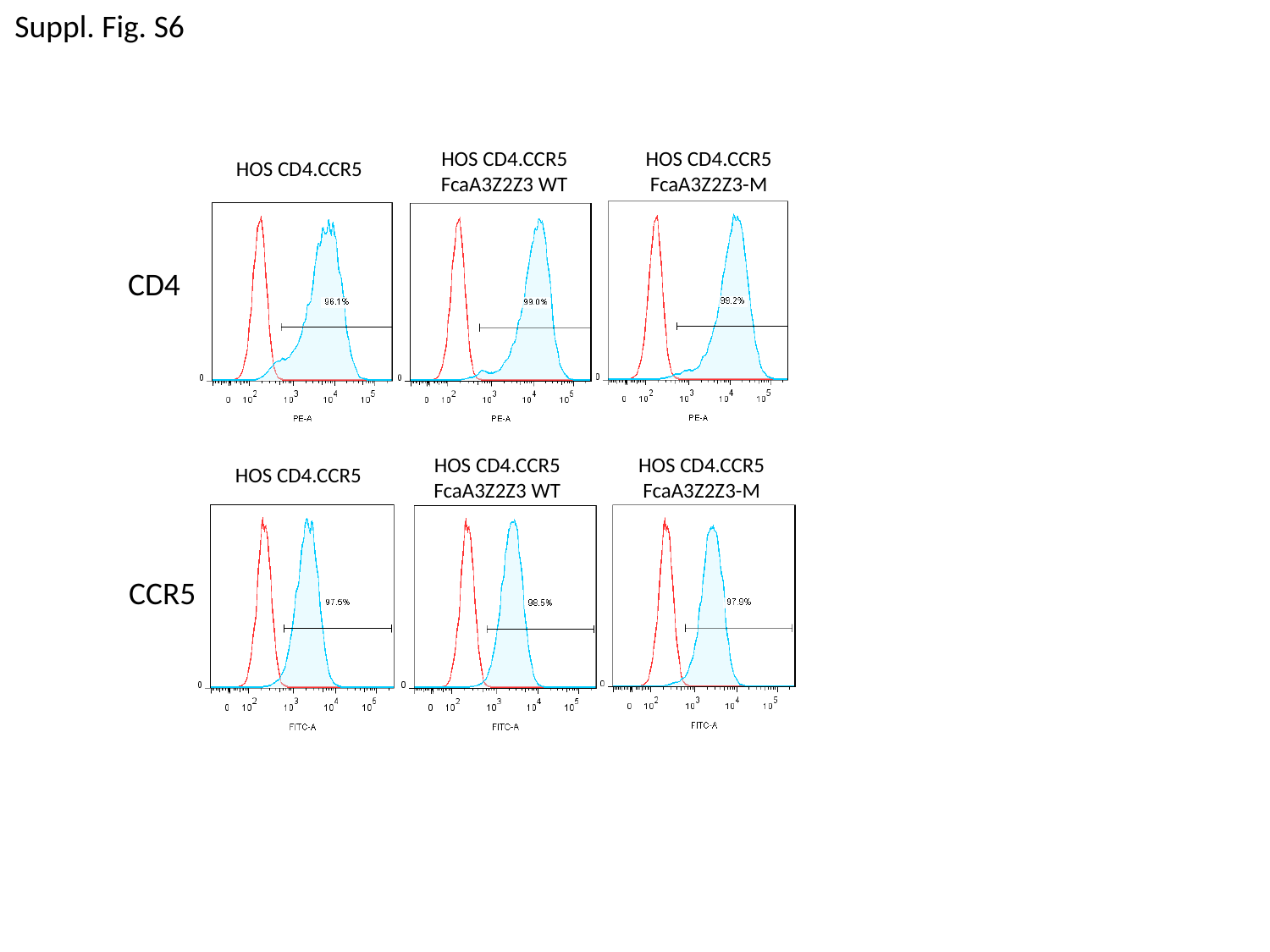

Suppl. Fig. S6
HOS CD4.CCR5
FcaA3Z2Z3 WT
HOS CD4.CCR5
FcaA3Z2Z3-M
HOS CD4.CCR5
CD4
HOS CD4.CCR5
FcaA3Z2Z3 WT
HOS CD4.CCR5
FcaA3Z2Z3-M
HOS CD4.CCR5
CCR5

## Slide 10
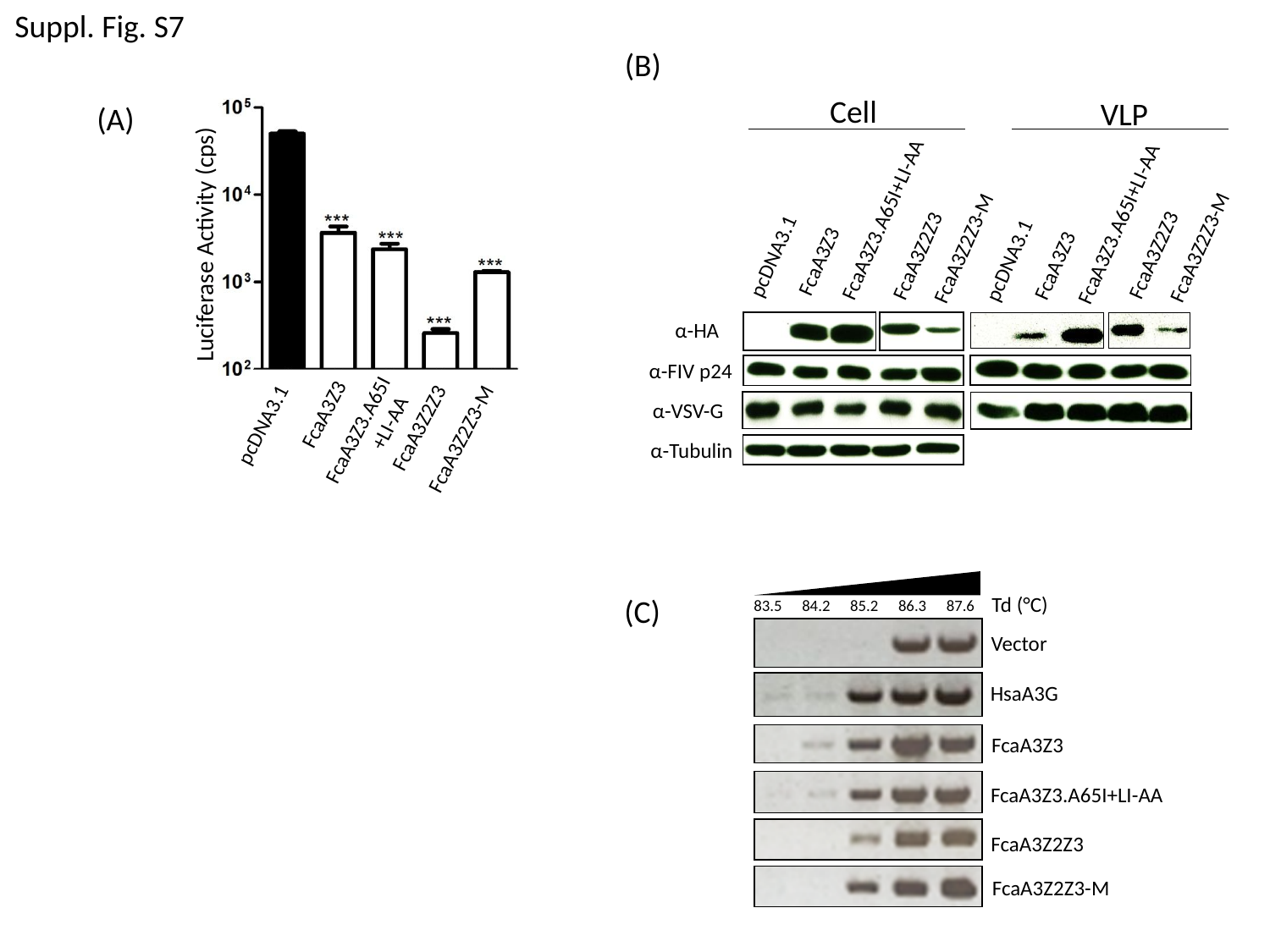

Suppl. Fig. S7
(B)
Luciferase Activity (cps)
pcDNA3.1
FcaA3Z3
FcaA3Z2Z3
FcaA3Z2Z3-M
Cell
VLP
(A)
FcaA3Z3.A65I+LI-AA
FcaA3Z3.A65I+LI-AA
FcaA3Z2Z3-M
FcaA3Z2Z3-M
FcaA3Z2Z3
FcaA3Z2Z3
FcaA3Z3
pcDNA3.1
FcaA3Z3
pcDNA3.1
α-HA
α-FIV p24
α-VSV-G
α-Tubulin
FcaA3Z3.A65I
+LI-AA
Td (°C)
(C)
83.5
84.2
85.2
86.3
87.6
Vector
HsaA3G
FcaA3Z3
FcaA3Z3.A65I+LI-AA
FcaA3Z2Z3
FcaA3Z2Z3-M

## Slide 11
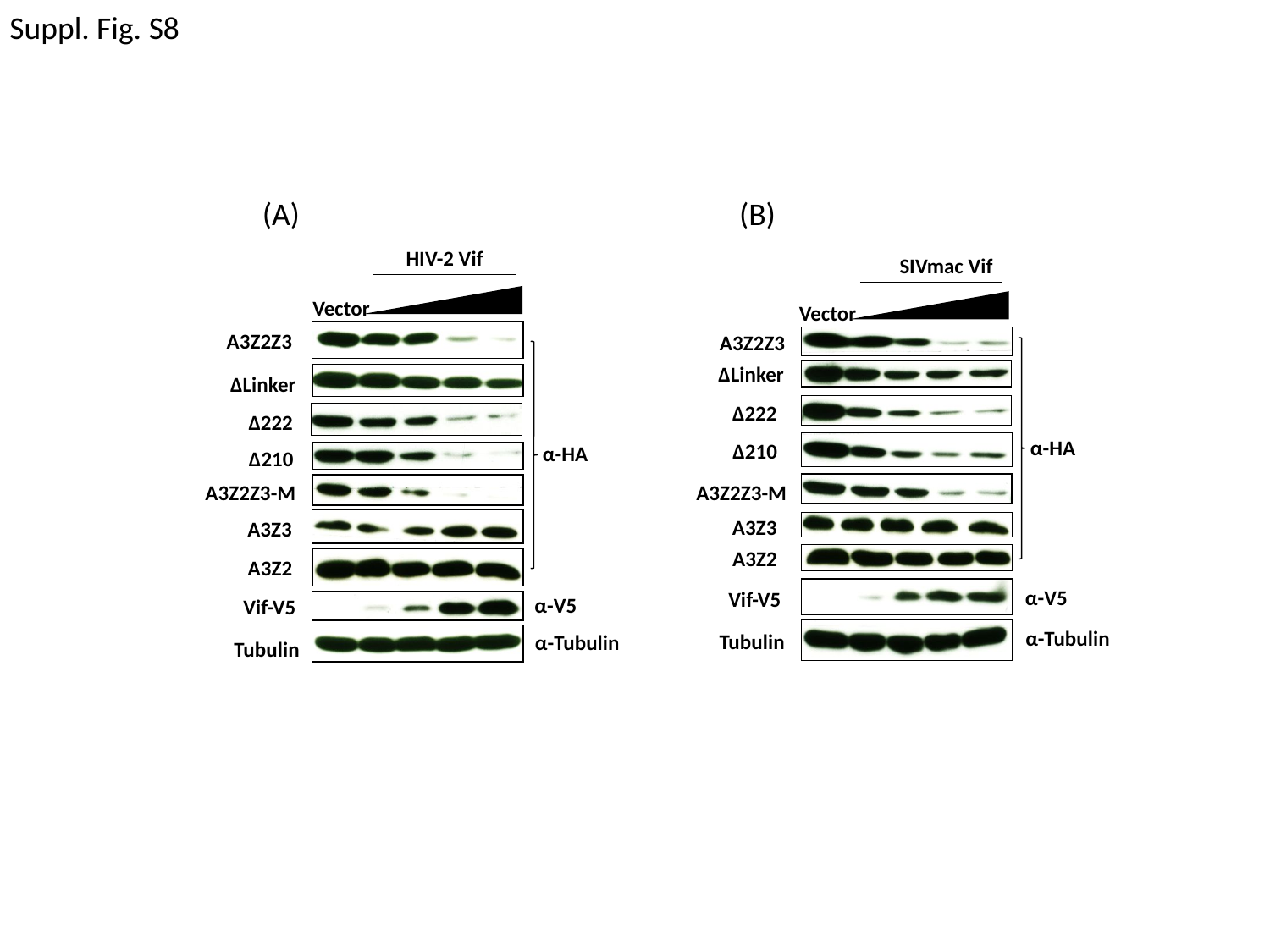

Suppl. Fig. S8
(B)
(A)
HIV-2 Vif
Vector
A3Z2Z3
ΔLinker
Δ222
α-HA
Δ210
A3Z2Z3-M
A3Z3
A3Z2
α-V5
Vif-V5
α-Tubulin
Tubulin
SIVmac Vif
Vector
A3Z2Z3
ΔLinker
Δ222
α-HA
Δ210
A3Z3
A3Z2
α-V5
Vif-V5
α-Tubulin
Tubulin
A3Z2Z3-M

## Slide 12
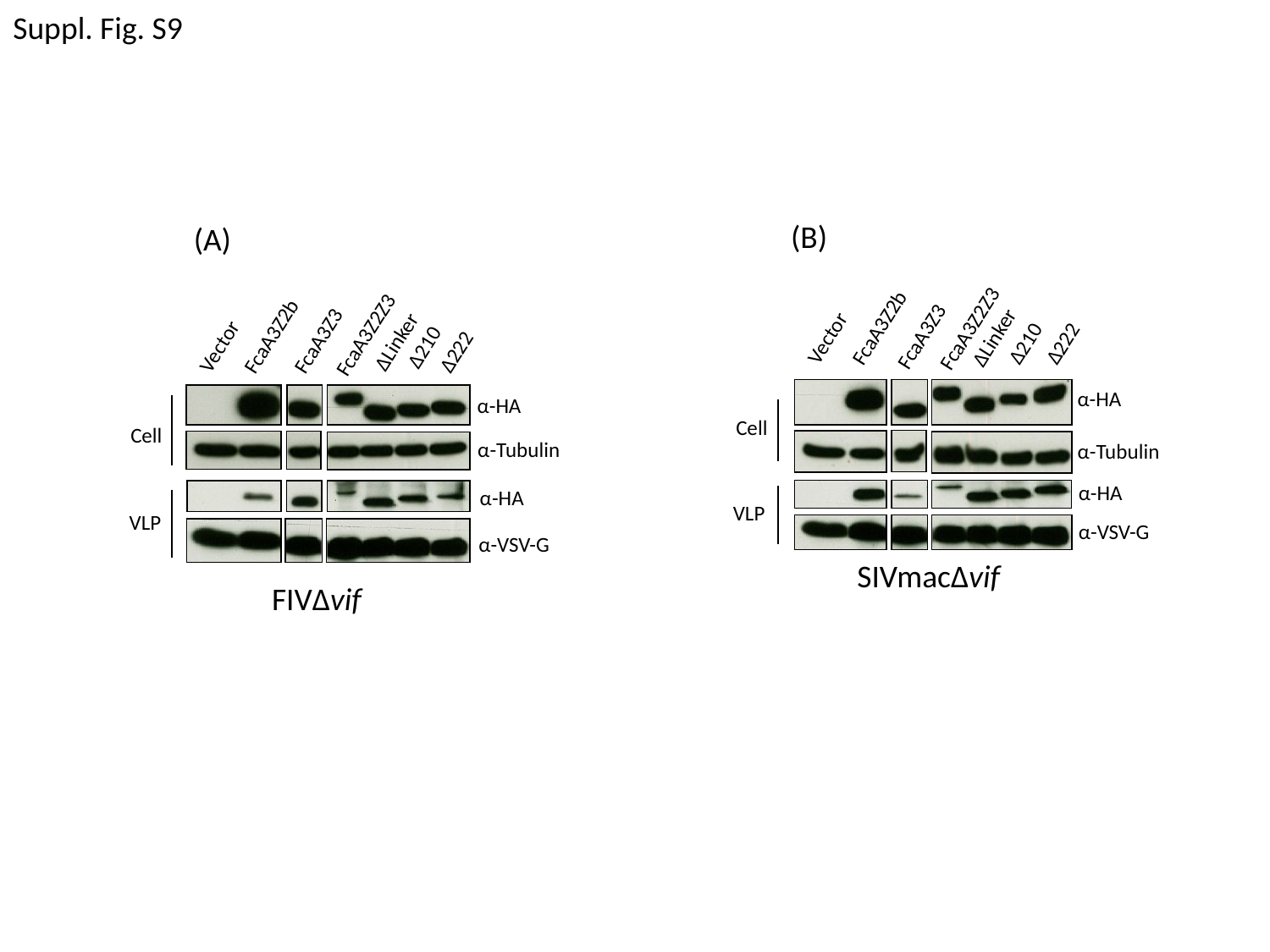

Suppl. Fig. S9
(B)
(A)
FcaA3Z2b
FcaA3Z2Z3
FcaA3Z3
ΔLinker
Vector
Δ222
Δ210
α-HA
Cell
α-Tubulin
α-HA
VLP
α-VSV-G
SIVmacΔvif
FcaA3Z2Z3
FcaA3Z2b
FcaA3Z3
ΔLinker
Vector
Δ210
Δ222
α-HA
Cell
α-Tubulin
α-HA
VLP
α-VSV-G
FIVΔvif

## Slide 13
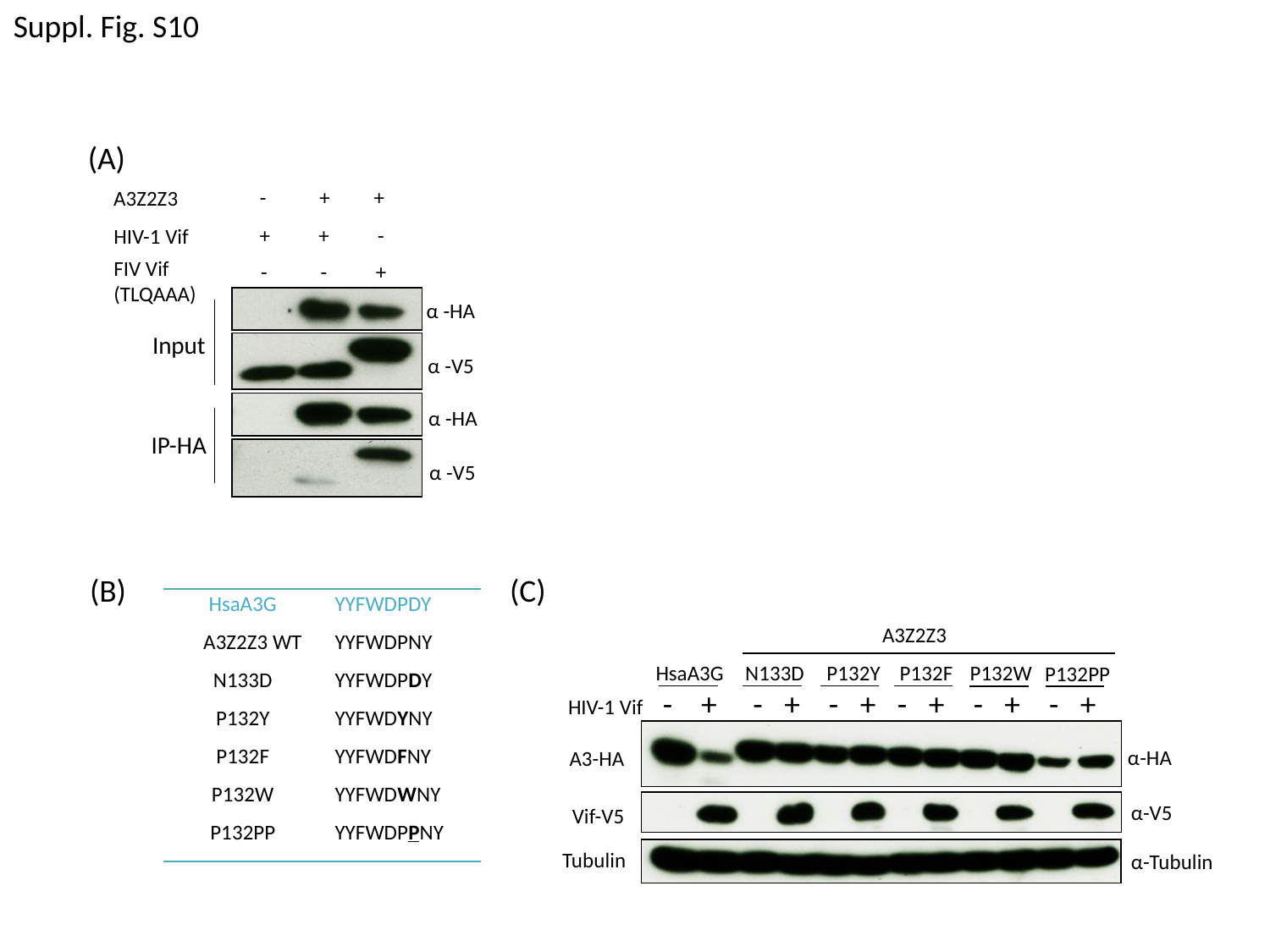

Suppl. Fig. S10
(A)
 - + +
A3Z2Z3
 + + -
HIV-1 Vif
FIV Vif
(TLQAAA)
 - - +
α -HA
Input
α -V5
α -HA
IP-HA
α -V5
(B)
(C)
| HsaA3G | YYFWDPDY |
| --- | --- |
| A3Z2Z3 WT | YYFWDPNY |
| N133D | YYFWDPDY |
| P132Y | YYFWDYNY |
| P132F | YYFWDFNY |
| P132W | YYFWDWNY |
| P132PP | YYFWDPPNY |
A3Z2Z3
P132F
P132W
HsaA3G
P132Y
N133D
P132PP
- + - + - + - + - + - +
HIV-1 Vif
α-HA
A3-HA
α-V5
Vif-V5
Tubulin
α-Tubulin
