# Supplementary material for: Determinants of FIV and HIV Vif sensitivity of feline APOBEC3 restriction factors
Source: Retrovirology. 2016 Jul 1;13:46. doi: 10.1186/s12977-016-0274-9 (PMC4930625; doi:10.1186/s12977-016-0274-9)
Supplement: Supplementary file 2 — 10.1186/s12977-016-0274-9. Primer list used for HsaA3C/FcaA3Z2 chimeras and FcaA3Z2 mutants. Table S2. Primer list used for HsaA3H/FcaA3Z3 chimeras and FcaA3Z3 mutants. Table S3. Primer list used for FcaA3Z2Z3 mutants. Table S4. Primer used to clone GST fusion constructs. Table S5. The software used in TopModel for threading, alignment and model quality estimationa. [file 12977_2016_274_MOESM2_ESM.docx]

**Supplemental Tables**

**Supplemental Table S1: Primer list used for HsaA3C/FcaA3Z2 chimeras and FcaA3Z2 mutants**

| **Construct** | **Primer Name** | **Primer Sequence** |
| --- | --- | --- |
| Z2C1 | fehuApo3 1-63.fw | 5’tataagctttgagagaggaatggagccctggcgcccagcccaagaaacccaatggacaggatagatcctaacaccttccgtttccaatttaaaaacctatg-3’ |
| Z2C4 | hufe3C 397.fw | 5’gcctctactacttctgggacccatgttaccaggaggggctccgcag-3’ |
|  | hufe3C 397.rv | 5’ctgcggagcccctcctggtaacatgggtcccagaagtagtagaggc-3’ |
| Z2C5 | hufe3C 493.fw | 5’aaacactgttgggacaactttgtgtacaatgataatgagccattcaa-3’ |
|  | hufe3C 493.rv | 5’ttgaatggctcattatcattgtacacaaagttgtcccaacagtgttt-3’ |
| Z2C30 | hufe3C 485.fw | 5’taaatattgttgggaaaactttgtggaccacaagggaatgcgctt-3’ |
|  | hufe3C 485.rv | 5’aagcgcattcccttgtggtccacaaagttttcccaacaatattta-3’ |
| FcaZ2bN18K | N18K.fw | 5’-gatagatcctaagaccttccgtttc-3’ |
|  | N18K.rv | 5’-gaaacggaaggtcttaggatctatc-3’ |
| FcaZ2bT44R | T44R.fw | 5’-cttccaagtggagagagaagactacttc-3’ |
|  | T44R.rv | 5’-gaagtagtcttctctctccacttggaag-3’ |
| FcaZ2bD165Y | D165Y.fw | 5’-caactttgtgtaccacaagggaatgc-3’ |
|  | D165Y.rv | 5’-gcattcccttgtggtacacaaagttg-3’ |
| FcaZ2bH166N | H166N.fw | 5’-caactttgtggacaacaagggaatgc-3’ |
|  | H166N.rv | 5’-gcattcccttgttgtccacaaagttg-3’ |
| FcaZ2b DH-YN | DH-YN.fw | 5’-caactttgtgtacaacaagggaatgc-3’ |
|  | DH-YN.rv | 5’-gcattcccttgttgtacacaaagttg-3’ |
| PtiZ2Y165D | Y165D.fw | 5’-caactttgtggaccacaagggaatgc-3’ |
|  | Y165D.rv | 5’-gcattcccttgtggtccacaaagttg-3’ |
| External primers | feApo3.fw | 5’tataagctttgaagaggaatggagccctggcgccccag-3’ |
|  | HA-rv | 5’agctcgagtcaagcgtaatctggaacatcgtatggataagcgtaatctggaacatcgtatg-3’ |

**Supplemental Table S2: Primer list used for HsaA3H/FcaA3Z3 chimeras and FcaA3Z3 mutants**

| **Construct** | **Primer Name** | **Primer Sequence** |
| --- | --- | --- |
| Z3C1 | Z3C1.fw | 5’ccagcaccgggtcccaaagccctactacccgaggaaggccctc-3’ |
|  | Z3C1.rv | 5’gagggccttcctcgggtagtagggctttgggacccggtgctgg-3’ |
| Z3C2 | Z3C2.fw | 5’caaagactgccttcgaaataagaaaaagtgccatgcagaaatttg-3’ |
|  | Z3C2.rv | 5’caaatttctgcatggcactttttcttatttcgaaggcagtctttg-3’ |
| Z3C6 | Z3C6.fw | 5’caagcgccgcctcagaaggccttactaccggaggaaaacctac-3’ |
|  | Z3C6.rv | 5’gtaggttttcctccggtagtaaggccttctgaggcggcgcttg-3’ |
| Z3C7 | Z3C7.fw | 5’gaggctactttgaaaacaagaaaaagcgccatgcggaaatgtg-3’ |
|  | Z3C7.rv | 5’cacatttccgcatggcgctttttcttgttttcaaagtagcctc-3’ |
| FcaZ3KL-TP | KL-TP.fw | 5’-gctaccagctgacgccgcccgaaggcacc-3’ |
|  | KL-TP.rv | 5’-ggtgccttcgggcggcgtcagctggtagc-3’ |
| FcaZ3PE-QN | PE-QN.fw | 5’-ccagctgaagctgcagaatggcaccctaattc-3’ |
|  | PE-QN.rv | 5’-gaattagggtgccattctgcagcttcagctgg-3’ |
| FcaZ3LI-TP | LI-TP.fw | 5’-gcccgaaggcaccacacctcacaaagactgcc-3’ |
|  | LI-TP.rv | 5’-ggcagtctttgtgaggtgtggtgccttcgggc-3’ |
| FcaZ3H-T | H-T.fw | 5’-cgaaggcaccctaattaccaaagactgcc-3’ |
|  | H-T.rv | 5’-ggcagtctttggtaattagggtgccttcg-3’ |
| FcaZ3DC-AA | DC-AA.fw | 5’-ctaattcacaaagccgcccttcgaaataag-3’ |
|  | DC-AA.rv | 5’-cttatttcgaagggcggctttgtgaattag-3’ |
| FcaZ3LR-AA | LR-AA.fw | 5’-cacaaagactgcgctgcaaataagaaaaag-3’ |
|  | LR-AA.rv | 5’-ctttttcttatttgcagcgcagtctttgtg-3’ |
| FcaZ3LI-AA | LI-AA.fw | 5’-gcccgaaggcaccgcagctcacaaagactgcc-3’ |
|  | LI-AA-rv | 5’-ggcagtctttgtgagctgcggtgccttcgggc-3’ |
| External primers | FcaZ3.fw | 5’-atgaattcgccaccatgaatccactacaggaag-3’ |
|  | HA-rv | 5’agctcgagtcaagcgtaatctggaacatcgtatggataagcgtaatctggaacatcgtatg-3’ |

**Supplemental Table S3: Primer list used for FcaA3Z2Z3 mutants**

| **Construct** | **Primer Name** | **Primer Sequence** |
| --- | --- | --- |
| Δ210 | Δ210.rv | 5’ctgtagtggattcattgtgggtctttgggcccctgggcggggagggaagggcc-3’ |
| Δ222 | Δ222.rv | 5’ctgtagtggattcattgtgggtctctctgtcacctcctgaacccaactccttggg-3’ |
| ΔLinker | Δlinker.fw | 5’gcttcaagaaatccttagacccacaatgaatccactacaggaag-3’ |
|  | Δlinker.rv | 5’cttcctgtagtggattcattgtgggtctaaggatttcttgaagc-3’ |
| FcaZ2Z3N133D | N133D.fw | 5’-ctacttctgggacccagattaccaggaggggc-3’ |
|  | N133D.rv | 5’-gcccctcctggtaatctgggtcccagaagtag-3’ |
| FcaZ2Z3P132Y | P132Y.fw | 5’-ctacttctgggactacaattaccaggaggggc-3’ |
|  | P132Y.rv | 5’-gcccctcctggtaattgtagtcccagaagtag-3’ |
| FcaZ2Z3P132F | P132F.fw | 5’-ctacttctgggacttcaattaccaggaggggc-3’ |
|  | P132F.rv | 5’-gcccctcctggtaattgaagtcccagaagtag-3’ |
| FcaZ2Z3P132W | P132W.fw | 5’-ctacttctgggactggaattaccaggaggggc-3’ |
|  | P132W.rv | 5’-gcccctcctggtaattccagtcccagaagtag-3’ |
| FcaZ2Z3P132PP | P132PP.fw | 5’-cttctgggacccaccaaattaccaggagg-3’ |
|  | P132PP.rv | 5’-cctcctggtaatttggtgggtcccagaag-3’ |

**Supplemental Table S4: Primer used to clone GST fusion constructs**

| **Construct** | **Primer Name** | **Primer Sequence** |
| --- | --- | --- |
| FcaGST-Z2-HA | FcaZ2b-GST-EcoRI-F | 5‘-ATAGAATTCCCatggagccctggcgcccc-3‘ |
|  | HA-NotI-R | 5‘-ATGCGGCCGCTCAAGCGTAATCTGGAACATC-3‘ |
| FcaGST-Z3-HA | FcaZ3-GST-EcoRI-F | 5‘-ATAGAATTCCCatgaatccactacaggaag-3‘ |
|  | HA-NotI-R | 5‘-ATGCGGCCGCTCAAGCGTAATCTGGAACATC-3‘ |
| FcaGST-Linker | Fca-Linker-EcoRI-F | 5‘-ATGAATTCCCagtcccggccaacaaag-3‘ |
|  | Fca-Linker-EcoRI-R | 5‘-ATGTCGACTCAtgtgggtctgggcaagag-3‘ |

**Supplemental Table S5:** **The software used in TopModel for threading, alignment and model quality estimation.^a^**

| **Threading** | **Alignment** | **Model Quality Estimation** |
| --- | --- | --- |
| **DeltaBLAST** [1] | **ClustalW*** [2] | **PROCHECK** [3] |
| **HMMER3** [4] | **POA*** [5] | **MolProbity** [6] |
| **HHblits** [7] | **MUSCLE*** [8] | **ANOLEA** [9] |
| **SAMT2K** [10] | **ProbA*** [11] | **ProSa2003** [12] |
| **FFAS03** [13] | **ProbCons*** [14] | **DOPE** [15] |
| **SPARKSX** [16] | **PCMA*** [17] | **GOAP** [18] |
| **RAPTORX** [19] | **DiAlign*** [20] | **ModFoldClust2** [21] |
| **LOMETS** [22] | **SAP*** [23] | **SPICKER** [24] |
|  | **TM-Align*** [25] |  |
|  | **MAFFT7** [26] |  |
|  | **MergeAlign2** [27] |  |
|  | **TCOFFEE** [28] |  |
|  | **PROMALS3D** [29] |  |
|  | **FORMATT** [30] |  |
|  | **MUSTANG** [31] |  |
|  | **3DCOMB** |  |
|  | **SALIGN** [32] |  |

^a^ Software marked with “*” are used within TCOFFEE.

References

1. Boratyn GM, Schaffer A, Agarwala R, Altschul SF, Lipman DJ, Madden TL: **Domain enhanced lookup time accelerated BLAST**. *Biol Direct* 2012, **7**(1):12.

2. Thompson JD, Higgins DG, Gibson TJ: **CLUSTAL W: improving the sensitivity of progressive multiple sequence alignment through sequence weighting, position-specific gap penalties and weight matrix choice**. *Nucleic acids research* 1994, **22**(22):4673-4680.

3. Laskowski RA, MacArthur MW, Moss DS, Thornton JM: **PROCHECK: a program to check the stereochemical quality of protein structures**. *Journal of applied crystallography* 1993, **26**(2):283-291.

4. Eddy SR: **Accelerated profile HMM searches**. *PLoS computational biology* 2011, **7**(10):e1002195.

5. Lee C, Grasso C, Sharlow MF: **Multiple sequence alignment using partial order graphs**. *Bioinformatics* 2002, **18**(3):452-464.

6. Chen VB, Arendall WB, Headd JJ, Keedy DA, Immormino RM, Kapral GJ, Murray LW, Richardson JS, Richardson DC: **MolProbity: all-atom structure validation for macromolecular crystallography**. *Acta Crystallographica Section D: Biological Crystallography* 2009, **66**(1):12-21.

7. Remmert M, Biegert A, Hauser A, Söding J: **HHblits: lightning-fast iterative protein sequence searching by HMM-HMM alignment**. *Nature methods* 2012, **9**(2):173-175.

8. Edgar RC: **MUSCLE: multiple sequence alignment with high accuracy and high throughput**. *Nucleic acids research* 2004, **32**(5):1792-1797.

9. Melo F, Feytmans E: **Novel knowledge-based mean force potential at atomic level**. *Journal of molecular biology* 1997, **267**(1):207-222.

10. Karplus K, Karchin R, Draper J, Casper J, Mandel‐Gutfreund Y, Diekhans M, Hughey R: **Combining local‐structure, fold‐recognition, and new fold methods for protein structure prediction**. *Proteins: Structure, Function, and Bioinformatics* 2003, **53**(S6):491-496.

11. Sierk ML, Smoot ME, Bass EJ, Pearson WR: **Improving pairwise sequence alignment accuracy using near-optimal protein sequence alignments**. *BMC bioinformatics* 2010, **11**(1):146.

12. Sippl MJ: **Recognition of errors in three-dimensional structures of proteins**. *Proteins: Structure, Function, and Genetics* 1993, **17**(4):355-362.

13. Rychlewski L, Li W, Jaroszewski L, Godzik A: **Comparison of sequence profiles. Strategies for structural predictions using sequence information**. *Protein Science* 2000, **9**(2):232-241.

14. Do CB, Mahabhashyam MS, Brudno M, Batzoglou S: **ProbCons: Probabilistic consistency-based multiple sequence alignment**. *Genome research* 2005, **15**(2):330-340.

15. Shen My, Sali A: **Statistical potential for assessment and prediction of protein structures**. *Protein science* 2006, **15**(11):2507-2524.

16. Yang Y, Faraggi E, Zhao H, Zhou Y: **Improving protein fold recognition and template-based modeling by employing probabilistic-based matching between predicted one-dimensional structural properties of query and corresponding native properties of templates**. *Bioinformatics* 2011, **27**(15):2076-2082.

17. Pei J, Sadreyev R, Grishin NV: **PCMA: fast and accurate multiple sequence alignment based on profile consistency**. *Bioinformatics* 2003, **19**(3):427-428.

18. Zhou H, Skolnick J: **GOAP: a generalized orientation-dependent, all-atom statistical potential for protein structure prediction**. *Biophysical journal* 2011, **101**(8):2043-2052.

19. Peng J, Xu J: **RaptorX: exploiting structure information for protein alignment by statistical inference**. *Proteins: Structure, Function, and Bioinformatics* 2011, **79**(S10):161-171.

20. Al Ait L, Yamak Z, Morgenstern B: **DIALIGN at GOBICS—multiple sequence alignment using various sources of external information**. *Nucleic acids research* 2013, **41**(W1):W3-W7.

21. McGuffin LJ, Roche DB: **Rapid model quality assessment for protein structure predictions using the comparison of multiple models without structural alignments**. *Bioinformatics* 2010, **26**(2):182-188.

22. Wu S, Zhang Y: **LOMETS: a local meta-threading-server for protein structure prediction**. *Nucleic acids research* 2007, **35**(10):3375-3382.

23. Taylor WR: **Protein structure comparison using iterated double dynamic programming**. *Protein Science* 1999, **8**(03):654-665.

24. Zhang Y, Skolnick J: **SPICKER: A clustering approach to identify near‐native protein folds**. *Journal of computational chemistry* 2004, **25**(6):865-871.

25. Zhang Y, Skolnick J: **TM-align: a protein structure alignment algorithm based on the TM-score**. *Nucleic acids research* 2005, **33**(7):2302-2309.

26. Katoh K, Standley DM: **MAFFT multiple sequence alignment software version 7: improvements in performance and usability**. *Molecular biology and evolution* 2013, **30**(4):772-780.

27. Collingridge PW, Kelly S: **MergeAlign: improving multiple sequence alignment performance by dynamic reconstruction of consensus multiple sequence alignments**. *BMC bioinformatics* 2012, **13**(1):117.

28. O'Sullivan O, Suhre K, Abergel C, Higgins DG, Notredame C: **3DCoffee: combining protein sequences and structures within multiple sequence alignments**. *Journal of molecular biology* 2004, **340**(2):385-395.

29. Pei J, Kim B-H, Grishin NV: **PROMALS3D: a tool for multiple protein sequence and structure alignments**. *Nucleic acids research* 2008, **36**(7):2295-2300.

30. Daniels NM, Nadimpalli S, Cowen LJ: **Formatt: Correcting protein multiple structural alignments by incorporating sequence alignment**. *BMC bioinformatics* 2012, **13**(1):259.

31. Konagurthu AS, Whisstock JC, Stuckey PJ, Lesk AM: **MUSTANG: a multiple structural alignment algorithm**. *Proteins: Structure, Function, and Bioinformatics* 2006, **64**(3):559-574.

32. Madhusudhan M, Webb BM, Marti-Renom MA, Eswar N, Sali A: **Alignment of multiple protein structures based on sequence and structure features**. *Protein Engineering Design and Selection* 2009, **22**(9):569-574.
